# Bayesian Structural Equation Envelope Model

**DOI:** 10.1017/psy.2025.10027

**Published:** 2025-08-08

**Authors:** Chuchu Wang, Rongqian Sun, Xiangnan Feng, Xinyuan Song

**Affiliations:** 1 Department of Statistics, The Chinese University of Hong Kong, Hong Kong, China; 2 School of Psychology, Shenzhen University, Shenzhen, China; 3 Department of Statistics and Data Science, Fudan University, Shanghai, China

**Keywords:** Bayesian approach, envelope model, factor analysis, structural equation model

## Abstract

The envelope model has gained significant attention since its proposal, offering a fresh perspective on dimension reduction in multivariate regression models and improving estimation efficiency. One of its appealing features is its adaptability to diverse regression contexts. This article introduces the integration of envelope methods into the factor analysis model. In contrast to previous research primarily focused on the frequentist approach, the study proposes a Bayesian approach for estimation and envelope dimension selection. A Metropolis-within-Gibbs sampling algorithm is developed to draw posterior samples for Bayesian inference. A simulation study is conducted to illustrate the effectiveness of the proposed method. Additionally, the proposed methodology is applied to the ADNI dataset to explore the relationship between cognitive decline and the changes occurring in various brain regions. This empirical application further highlights the practical utility of the proposed model in real-world scenarios.

## 1 Introduction

The envelope model is initially introduced by Cook et al. (2010) as a technique for dimension reduction in multivariate analysis. Cook et al. (2010) first proposed the response envelope model, which aims to reduce the dimension of the response vector 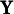 . The core concept of the method is to decompose 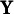 into a material and an immaterial part based on the assumption that certain linear combinations of 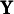 , known as X-invariant, remain unchanged regardless of variations in the predictor vector 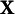 . These X-invariant linear combinations are considered immaterial to the regression and thus can reduce parameter space in estimation. Expanding upon this idea, Cook et al. (2013) extended the approach to include the dimensionality reduction in 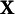 within the context of multivariate regression, assuming the predictor 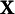 is stochastic. This extension, known as the predictor envelope model, is closely related to the partial least squares (PLS) method.

The envelope model has shown promising efficiency gains, which has prompted its extension into various contexts, including the partial envelope model (Su & Cook, 2011), generalized linear model (Cook & Zhang, 2015a), matrix-valued response model (Ding & Cook, 2018), sparse envelope model (Su et al., 2016) and spatial envelope model (Rekabdarkolaee et al., 2020). However, applying Bayesian methods to the envelope model has received limited attention compared to the frequentist framework in previous literature. This fact is primarily due to the challenge posed by parameterizing the envelope subspace within the Grassmann manifold space, where the basis of the envelope subspace is not unique. Nevertheless, considering the envelope model from a Bayesian perspective is highly meaningful, as it allows for incorporating prior information into posterior inference without relying on asymptotic assumptions.

Regarding Bayesian envelope models, Khare et al. (2017) first proposed a Bayesian approach for analyzing response envelope models. They reparameterized the envelope model in a Steifel manifold to ensure the uniqueness of the orthogonal basis and adopted the Bingham distribution as a prior for the orthogonal basis matrix. However, this method is developed based on a specific design of the response envelope model, and it may lose parameter conjugacy in other scenarios. Additionally, the implementation of this method relies on sampling from the matrix Bingham distribution and the truncated inverse Gamma distribution, which can result in a heavy computational burden. On the other hand, Cook et al. (2016) developed a novel parameterization for the orthogonal basis matrix of the envelope subspace, and this idea was further extended to the Bayesian framework by Chakraborty & Su (2024). The proposed method does not rely on the Grassmann or Steifel manifold and can be applied to various envelope model contexts, including the response and predictor envelopes. Meanwhile, Lee et al. (2022) adopted the same technique and formulated the envelope model for Bayesian quantile regression.

Inspired by the successful application of the reparameterized envelope method, this study introduces a factor analytic technique to the predictor envelope model, enabling dimension reduction for both the response and predictor variables. Factor models, such as confirmatory factor analysis (CFA) and exploratory factor analysis (EFA), are widely utilized statistical tools in fields such as psychology, education, and social sciences. Over the past few decades, existing studies have demonstrated the effectiveness of the factor model in capturing latent structures and reducing dimensionality by summarizing the latent factors through multiple observed variables. Moreover, the joint modeling approach, also known as the structural equation model (SEM), has shown high potential for adapting to various modeling techniques. The joint model typically consists of two parts. The first part entails a factor analysis model aggregating multiple observed variables into latent factors. This step captures the underlying structure and interrelationships among the observed variables. The second part utilizes a regression model to elucidate the association between the latent factors and the observed covariates of interest. It allows for adapting various modeling techniques, enabling a flexible and versatile analysis. For example, Roy & Lin (2000) used multiple longitudinal measures as outcomes to quantify a latent variable of interest from different perspectives. They adopted a linear mixed model to study the effects of covariates on the time-dependent latent variable. Pan et al. (2019) integrated latent variables into a proportional hazards model to examine the observed and latent risk factors associated with the failure time of interest. Wang et al. (2021) introduced a latent-on-image model to jointly analyze high-dimensional imaging data and multiple clinical measurements in an Alzheimer’s disease (AD) study. They characterized the severity of AD using various cognitive test scores as a latent factor in a CFA model and investigated the relationship between changes in brain structure and cognitive decline using a functional data regression model. Besides, researchers have developed plenty of methods to handle flexible data structure with a factor model, including hierarchical and heterogeneous data (Lee & Song, 2004), missing data (Song & Lee, 2006), longitudinal data (Song et al., 2011).

Bayesian methods have been extensively applied to SEM topics because they emphasize individual-level random observations and the estimation of first-order moment properties. This approach offers a simpler alternative to the traditional approach of fitting the covariance structure. By focusing on the raw individual-level data, Bayesian methods provide a flexible and intuitive framework for specifying prior distributions, incorporating prior knowledge, and conducting posterior inference. Furthermore, the hierarchical representation of the model, combined with efficient Markov chain Monte Carlo (MCMC) algorithms, allows for a straightforward statistical inference and accommodates highly complex models. For instance, Wang et al. (2016) integrated a mixture representation of the quantile regression model (Kozumi & Kobayashi, 2011; Yu & Moyeed, 2001) into SEM, moving beyond the usual assumption of normal errors. Feng et al. (2017) incorporated the Bayesian version of Lasso (BLasso) and adaptive Lasso (BaLasso) to quantile SEM, enabling simultaneous estimation and variable selection in this context.

This study is motivated by a real-world data analysis of AD. A comprehensive understanding of the relationship between changes in brain structure, cognitive function, and the progression of brain disease is critical for accurate diagnosis and prevention of brain-related disorders. In the context of brain degeneration, it is often a global process that affects multiple regions rather than being confined to specific areas. Therefore, understanding the interdependencies among different regions of interest (ROIs) is crucial. The Envelope method, which we employed in this study, allows us to investigate and uncover these interdependencies among ROIs. By examining the relationships among various brain regions, we aim to identify the most informative ROI or combination of ROIs that can provide valuable insights into the underlying mechanisms of brain diseases.

The main contribution of this article is to formulate a novel envelope approach within the context of Bayesian SEM. Introducing the envelope model to the structural component of SEM serves a dual purpose. On the one hand, the envelope model eliminates immaterial variation in the data, thereby enhancing estimation efficiency. This enhancement is particularly valuable when confronted with high-dimensional candidate predictors (e.g., neuroimaging phenotypes derived from MRI data), of which only a small subset are genuinely influential so that high-dimensional predictors can be projected to a reduced subspace. On the other hand, grouping latent variables from multiple observed surrogates also represents a form of dimension reduction, especially pertinent in situations, where the correlation among multivariate responses arises from the shared underlying mechanism of reflecting the same latent construct from varying perspectives (e.g., cognitive impairment is manifested by multiple cognitive tests together in the ADNI study). Therefore, the proposed Bayesian Envelope SEM (BESEM) offers a novel perspective on concurrent dimension reduction for both response variables and predictors. This is achieved by employing factor analysis in the domain of latent variable modeling for the former and integrating an envelope structure into the structural equation for the latter. Even in scenarios, where a lower-dimensional envelope subspace does not exist, the proposed method seamlessly degenerates to a standard SEM without compromising parameter estimation accuracy. To our knowledge, this work is the first to introduce envelope methods to the SEM framework. We restrict the estimation of the envelope space to an orthogonal basis, which greatly reduces the computation efficiency. The derived posterior distributions are proved to be proper even with non-informative priors. A simple block Metropolis-within-Gibbs MCMC algorithm is presented to facilitate posterior sampling. The proposed Markov chain is shown to be 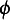 -irreducible and aperiodic, ensuring the convergence of MCMC samples.

The remainder of the article is organized as follows. Section 2 outlines the envelope model and defines BESEM. Section 3 discusses the Bayessian inference. Section 4 presents simulated experiments, showing the efficiency gains of BESEM compared to conventional Bayesian SEM approaches. In Section 5, we apply BESEM to the Alzheimer’s Disease Neuroimaging Initiative (ADNI) study to explore new insights into the relationship between cognitive decline and different brain regions. Technical details are provided in the Supplementary Material.

## 2 Model description

### 2.1 Review of envelope model

In this section, we provide a brief overview of the envelope idea proposed by Cook et al. (2010). This idea was initially developed to reduce the regression coefficients in the multivariate linear regression model given by (1)

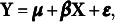

where 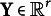 , 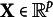 represent the response and predictor vector, respectively, 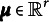 and 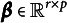 are the intercept and coefficients, and 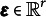 is the error term with a zero mean and a positive definite covariance matrix 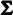 .

The response envelope model aims to decompose the response variable 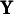 into a material part and an immaterial part based on the assumption that X-invariant linear combinations of 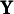 exist. Specifically, let 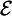 be a subspace of 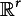 , 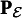 is the projection onto 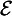 and 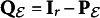 , where 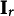 is the identity matrix of size 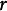 . The response envelope seeks to find the minimal subspace 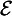 that satisfies the following two conditions:

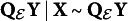
 , where 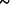 denotes equality in distribution and

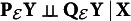
 .

These two conditions stipulate that the distribution of 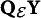 is not influenced by 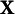 nor by 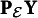 , indicating that the dependence of 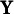 on 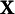 is concentrated only in 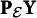 , which is referred to as the material part. Then, 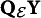 is the immaterial part. Cook et al. (2010) showed that (i) and (ii) are equivalent to the following two conditions:

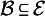
 , where 
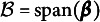
 and

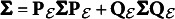
 .

Condition (ii 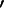 ) states that 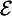 is a reducing subspace of 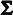 . Combined with condition (i 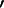 ), the 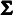 -envelope of 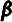 , denoted by 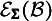 , is defined as the smallest reducing subspace of 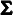 that contains 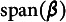 . The existence of the 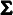 -envelope of 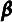 was discussed by Cook et al. (2010).

Let 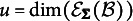 , 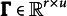 and 
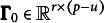
 be the orthogonal bases of 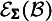 and 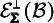 , respectively. Here, 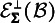 is the orthogonal complement of 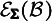 . Model (1) can be parameterized in terms of 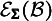 as follows: (2)

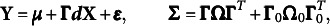

where 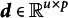 is the coordinates of 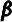 with respect to 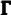 , and we have 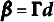 . The matrices 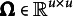 and 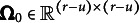 are positive definite and specify the covariance structure of the material and immaterial parts. For a fixed 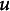 , the total number of parameters required for the model (2) is 

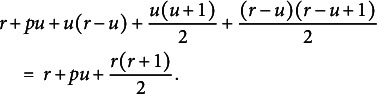

If 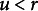 , the efficiency gains of the envelope are possible compared to the standard multivariate linear regression model (1). If 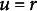 , the envelope model degenerates into the linear regression standard model.

The predictor envelope model is built on a framework similar to the response envelope model, aiming to reduce the dimensionality of 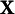 . To accommodate a stochastic predictor 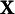 with mean 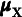 and variance 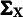 , model (1) is modified as follows: (3)

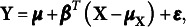

where 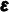 is independent of 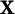 and not necessarily normally distributed. To decompose 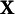 into its material and immaterial parts, we assume there is a subspace 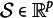 that satisfies the following conditions:

- cov 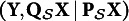 = 0 and
- cov 
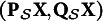
 = 0.

These conditions indicate that 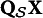 is uncorrelated with both 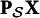 and 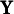 , while all the information in 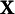 that is linearly related to the regression is captured by 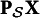 . It has been shown that these conditions are equivalent to the following ones (Cook et al., 2013):

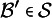
 , and

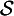
 is a reducing subspace of 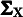 ,

where 
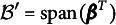
 . The intersection of all subspaces satisfying these two properties is referred to as the 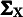 -envelope of 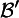 , denoted as 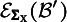 . Let 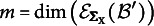 , and 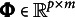 be a orthogonal basis of 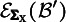 . Then, the predictor envelope model is formulated as (4)

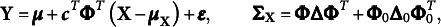

where 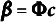 , 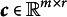 is the coordinate of 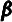 with respect to 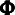 , 
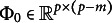
 is an orthogonal basis of 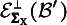 , and 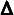 and 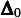 are positive definite matrices.

Considering that reducing the dimensionality of complex covariates is often of great interest in efficiently quantifying the relationship between the latent outcomes and a set of predictors in SEM, in the following section, we focus on the aspect of predictor dimension reduction to describe the proposed methodology.

### 2.2 BESEM

We introduce the envelope approach in the context of SEM. Let 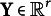 be the observed response vector like the previous representation. In addition, we let 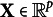 be the vector of random predictor variables with mean 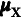 and variance 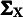 , and 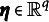 be a vector of latent variables that are expected to be formulated from the observed variables in 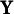 . A standard SEM can be defined as follows: (5)

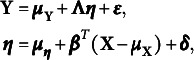

where 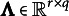 is the unknown factor loading matrix, 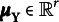 and 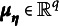 are intercepts, 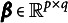 is the unknown coefficient of interest, and 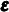 and 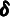 are independent error terms. We assume 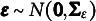 and 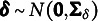 , where 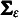 is a diagonal matrix with diagonal elements 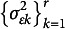 , and 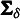 is a positive definite matrix.

The first equation in (5) represents the link between the observed outcome variables 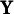 and the latent variables 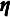 , characterized by a CFA model. The second equation in (5) is a structural equation to assess the effects of the covariates of interest 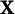 on 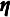 .

To reduce the dimensionality of 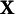 with an envelope structure and decompose 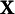 into its material and immaterial parts, we assume there is a subspace 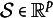 that satisfies the following conditions:

- cov 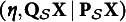 = 0 and
- cov 
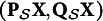
 = 0.

These conditions indicate that 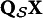 is uncorrelated with both 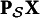 and 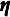 , while all the information in 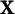 that is linearly related to 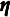 in the SEM is captured by 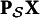 . The conditions (a) and (b) are equivalent to the following two conditions (Cook et al., 2013):

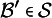
 , where 
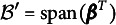
 and

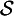
 is a reducing subspace of 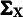 .

Therefore, we aim to find the smallest subspace that satisfies (a 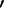 ) and (b 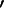 ), or equivalently, the intersection of all reducing subspaces of 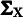 that contains 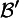 , which is the 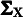 -envelope of 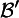 , denoted as 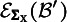 .

Let 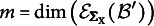 , and 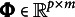 be a orthogonal basis of 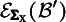 . Then, the SEM model (5) can be formulated as the following envelope SEM: (6)

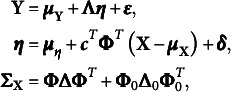

where 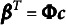 , 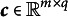 is the full rank coordinate of 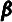 with respect to 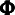 , 
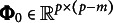
 is an orthogonal basis of 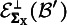 . The matrices 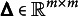 and 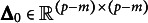 are positive definite. When 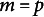 , the proposed envelope model is equivalent to a standard SEM.

### 2.3 Reparametrization of BESEM

For a fixed 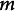 , the envelope 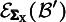 is defined on a 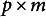 Grassmann manifold, which implies that the specification of the basis 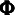 is not unique. We can reparameterize the envelope model to address this issue and identify a unique orthogonal basis in an Euclidean space (Cook et al., 2016).

Let 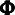 be an arbitrary basis of 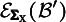 . We define 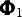 as the first m rows of 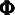 . Without loss of generality, we assume that 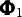 is nonsingular (otherwise, we can reorder the rows in 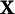 ). The remaining 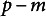 rows of 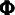 are denoted as 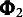 . 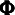 can be expressed as follows: (7)

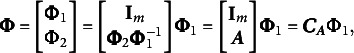

where 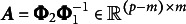 is an unconstrained matrix, and 
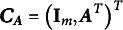
 is also a basis of 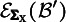 . It has been shown that 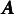 depends on 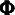 only through 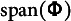 : suppose 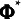 is a different basis of 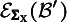 and 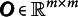 is a full rank matrix, such that 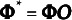 , then 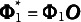 , 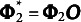 , and 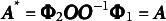 (Su et al., 2016). Therefore, there is a one-to-one correspondence between 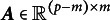 and 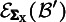 . Furthermore, the orthonormal basis of 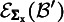 can be expressed as 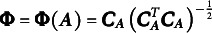 .

Based on (Chen et al., 2020, Lemma 1), we can construct a basis of 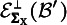 by forming the matrix 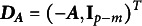 . Likewise, we can obtain an orthonormal basis as 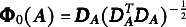 . Accordingly, (5) can be written as (8)

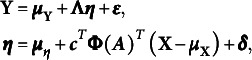

where 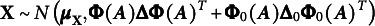 .

### 2.4 Model identification

The measurement equation in (8) is not identified because, for any nonsigular matrix 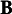 , we have 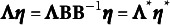 , where 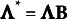 , and 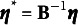 is still random latent variables. Imposing identification constraints on the measurement equation is necessary to establish identification. One commonly used approach, as described in Song & Lee (2012), is to define 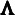 in a non-overlapping structure with a fixed nonzero element in each column. Here is an illustrative example: consider a scenario with 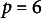 observed variables and 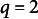 latent variables. In this case, the first four observed variables are associated with the first latent factor, while the remaining two are related to the second latent factor. Let 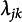 denote the 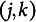 th element of 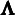 . A non-overlapping structure of 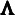 can be defined as follows: 

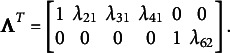



In the above structure, the elements with values 1 and 0 are known parameters with fixed values, while the 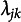 s’ represent the unknown parameters that need to be estimated. Therefore, the total number of parameters that need to be estimated is 

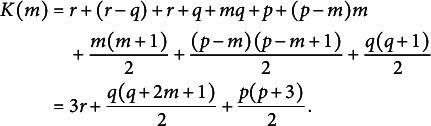



### 2.5 Envelope dimension selection

Determining the optimal dimension of the envelope space is an essential step before estimating the envelope model. Various information criteria, such as the Akaike information criterion (AIC), Bayesian information criterion (BIC), and the deviance information criteria(DIC), have been widely used in previous research. Information criteria offer a trade-off between model fit and complexity, with lower values indicating better-fitting models. The dimension of the envelope space that minimizes the AIC, BIC, or DIC can be chosen as the optimal dimension. However, there is no universally applicable guideline regarding which information criterion performs best in all scenarios. The performance of these criteria may vary depending on the specific characteristics of the data and the model under consideration. Prior studies, such as Shen et al. (2023) and Khare et al. (2017); and Chakraborty & Su (2024), have demonstrated that different information criteria have distinct performance in various envelope model contexts. Therefore, we investigate the performance of several information criteria, including AIC, BIC, DIC, and average weighted estimation (AWE), in determining the optimal dimension of the envelope space in the context of BESEM by conducting an empirical experiment in Section 4. Let 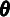 be all the unknown parameters and 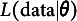 be the likelihood, then 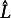 is the maximized likelihood value of the model. The deviance is 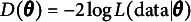 . The values of AIC, BIC, DIC, and AWE are calculated as follows: 

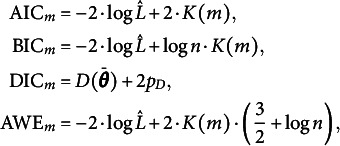

where 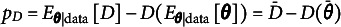 denotes the posterior mean deviance minus the deviance evaluated at the posterior mean of the parameters.

This empirical analysis provides valuable insights into the relative performance of information criteria and their suitability in specific scenarios of envelope models.

## 3 Bayesian inference

### 3.1 Prior distributions

Let 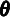 represent all the unknown parameters in model (8), and 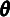 = 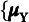 , 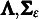 , 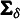 , 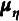 , 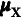 , 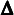 , 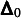 , 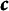 , 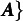 . We define a joint prior density 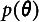 for the unknown parameters, which can be decomposed as 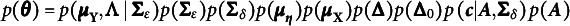 .

We first specify some notations: 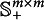 denotes the set of 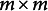 symmetric positive definite matrices, 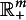 denotes the set of vectors of length 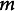 with positive values, 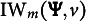 represents the Inverse-Wishart distribution with scale matrix 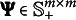 and degree of freedom 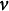 , 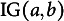 represents the Inverse-Gamma distribution, and 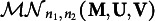 is the matrix normal distribution with the parameters 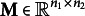 , 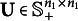 , 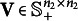 .

The priors for the concerned parameters are defined as follows:

- Let 
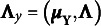
 , then for each 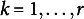 , where 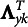 represents the 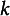 th row of 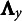 , we assign a joint prior to 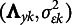 , given by 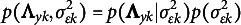 . Specifically, 

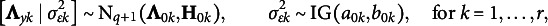

where 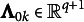 , 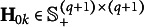 , 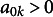 and 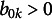 are prefixed hyperparameters.
- We adopt flat priors for the intercept terms 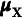 and 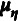




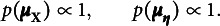

- We assign the Inverse-Wishart distribution to 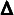 , 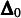 , and 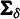 : 

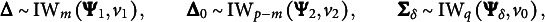

where 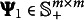 , 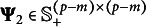 , 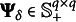 , 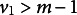 , 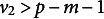 , and 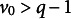 , are hyperparameters.
- We use matrix normal prior for 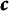 and 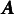 : 

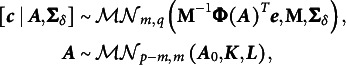

where 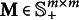 , 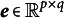 , 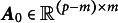 , 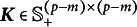 , 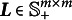 are hyperparameters.

Vague (noninformative) priors are used in the numerical studies. Such vague priors are appropriate choices widely adopted when limited information is available about the relationship between the latent responses and a large number of covariates. However, the proposed method can also straightforwardly accommodate prior knowledge once available. For instance, if we have prior information on the factor loading matrix 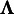 in the form of 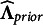 , we can set the prior mean of the loading matrix as 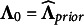 with a relatively small prior variance matrix 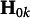 . Similarly, if we possess prior knowledge on the potential envelope subspace, i.e., 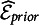 , we can determine the prior information for 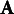 , 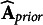 , through the one-to-one correspondence in Equation (7) and set 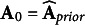 . In line with the methodology proposed by Chakraborty & Su (2024), even partial prior information concerning 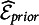 can be incorporated into the proposed method. For example, assuming a candidate envelope dimension of 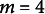 and we only know about 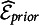 that it contains two independent unit vectors 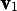 and 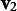 . In such circumstances, we can generate two random vectors from span 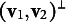 as 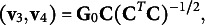 where 
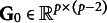
 is an orthonormal basis of span 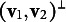 and 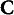 is a 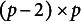 matrix with each entry independently generated from the standard normal distribution. Subsequently, the prior orthonormal basis can be formulated as 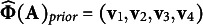 and 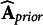 can be derived through Equation (7).

### 3.2 Posterior analysis and sampling process

Let 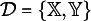 represent the collection of 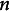 independent observations of 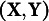 , where 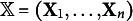 and 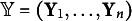 . Additionally, let 
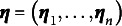
 denote the matrix of latent variables. The Bayesian estimate of 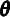 is commonly defined as the sample mean or mode of the posterior distribution 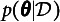 . However, drawing samples from 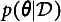 can be challenging due to the presence of the latent variable 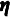 , as 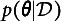 may not have a closed form. We utilize the data augmentation technique proposed by Tanner & Wong (1987) to address this issue. In this approach, we treat the latent variables 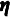 as missing data and augment the observed data with them. Consequently, the posterior sampling procedure can be constructed based on the complete data set and the joint distribution of 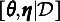 . Theorem 1 establishes the propriety of the posterior density, and its proof is included in the Supplementary Material.

Theorem 1 **(Posterior propriety)**

The posterior density 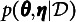 is proper with respect to Lebesgue measure on 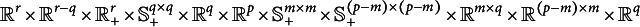 .

Given the complexity of the posterior distribution 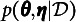 and the challenge of directly sampling from it, we propose a Metropolis-within-Gibbs sampler to draw posterior samples. The Gibbs sampler allows us to generate samples for each element of 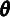 and 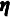 from their respective full conditional distributions iteratively. The proposed MCMC sampler (Algorithm 1) for the case when envelope dimension 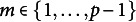 can be found in the Supplementary Material.

We also prove that the Markov chain generated by the proposed algorithm is 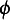 -irreducible and aperiodic in Theorem 2, which ensures the convergence to its stationary distribution from almost all starting states (Cinlar, 2013).

Theorem 2 ( 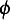 -irreducible and aperiodic)

The Markov chain generated by the Metropolis-within-Gibbs algorithm for the posterior sampling is 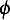 -irreducible and aperiodic.

## 4 Simulation

We design a simulation experiment to assess the performance of the BESEM methodology proposed in this article. The data are generated using equations (7) and (8). We consider three dimensions of the underlying envelope space, denoted as 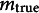 , which include values of 2, 4, and 6. Additionally, we consider two different settings for the predictor dimension 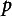 , with values of 20 and 40. The other parameters are fixed as follows: 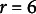 and 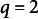 . To ensure identification, 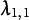 and 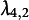 are set to 1, while the remaining free elements of 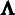 are generated independently from a uniform distribution 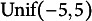 . The elements of 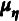 , 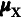 , and 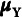 are independently sampled from a uniform distribution 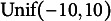 , and the elements of 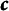 and 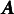 are drawn independently from uniform distributions 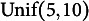 and 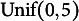 , respectively. The matrices 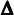 , 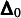 and 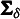 are generated independently from 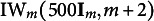 , 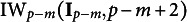 , and 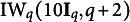 , respectively. The diagonal elements of the error covariance matrix 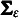 are simulated from 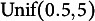 . More specifically, we first generate 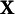 from a multivariate normal distribution characterized by parameters 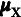 , 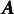 , 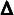 , and 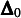 as mentioned before in Section 2.3. The error terms 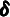 and 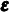 are independently drawn from normal distributions with zero mean and covariance matrix 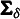 and 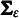 , respectively. The latent variables 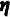 and the corresponding observed responses 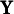 are then generated according to Equation (8). The simulation experiment considers different sample sizes, namely, 50, 150, 300, and 600. For each sample size, 100 replicated datasets are generated.

**Table 1:**
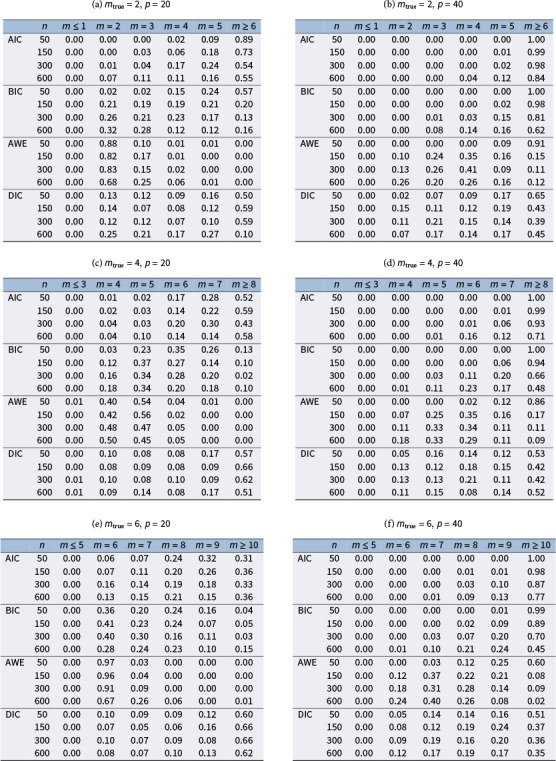
Percentages of estimated envelope dimension 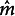 that were selected by the AIC, BIC, AWE, and DIC methods in 100 replications

For model estimation and Bayesian inference, we consider vague priors on the unknown parameters as follows:

1. For the prior distributions of 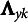 and 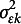 , we set 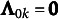 , 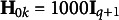 , 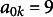 , 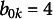 , for 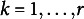 .
2. For the prior distributions of 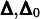 and 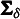 , the scale matrices 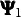 , 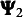 , 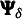 of the Inverse-Wishart distributions are set to 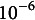 times the identity matrix. The degrees of freedom are set to 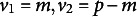 , and 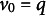 .
3. For the prior distributions of 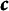 and 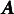 , the prior covariance matrices are specified as 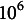 times the identity matrix, and the prior means are set to zero.

We use the trace plots of three Markov chains starting from different initial values to check convergence of the algorithm. Figure S1 in the Supplementary Material depicts the trace plots of several randomly selected elements of 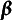 , showing that the Markov chains mixed well within the initial several thousand iterations. Therefore, we run the MCMC algorithm for 16000 iterations, with the first 8000 iterations as the burn-in stage. Four information criteria, AIC, BIC, DIC, and AWE, are employed for model selection. Due to the presence of the latent factor, the complete likelihood can be represented as an integration of 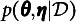 with respect to 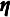 , which can be complex. We use the importance sampling approach to approximate the maximized complete data likelihood. In each replication, we calculate the information criteria for different values of m ranging from 0 to 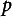 . The case where 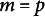 corresponds to the standard SEM method. The estimated envelope dimension, denoted as 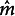 , is selected based on the minimum criterion value.

The accuracy of the model selection is assessed by determining the percentage of correctly identifying the true envelope dimension 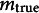 . The selection rate results for the different settings of 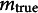 and 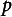 are presented in Table 1. As the sample size 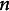 increases, the selected 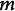 tends to concentrate more on the true envelope dimension 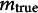 for all methods. The selection accuracy varies across different settings of 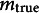 , 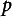 , and the true parameter value, and all four methods can exhibit good or poor performance in certain situations. However, among the four methods compared, the AWE method consistently achieves the highest accuracy across all settings. It is important to note that all four methods tend to overestimate the true envelope dimension. This overestimation has also been observed in previous studies (Chakraborty & Su, 2024; Lee et al., 2022). In such cases, the cost of overestimating 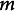 may involve losing some efficiency gained from dimension reduction, yet without introducing any bias in parameter estimation, as no material information is lost. The encouraging finding is that despite the fluctuating and dissatisfying selection rate, the overestimated models selected by AWE method still perform comparably to the true envelope model, although there might be a slight loss in efficiency. This assertion is supported by the estimation results of 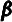 , which is one of the main focuses of this analysis.

**Figure 1:**
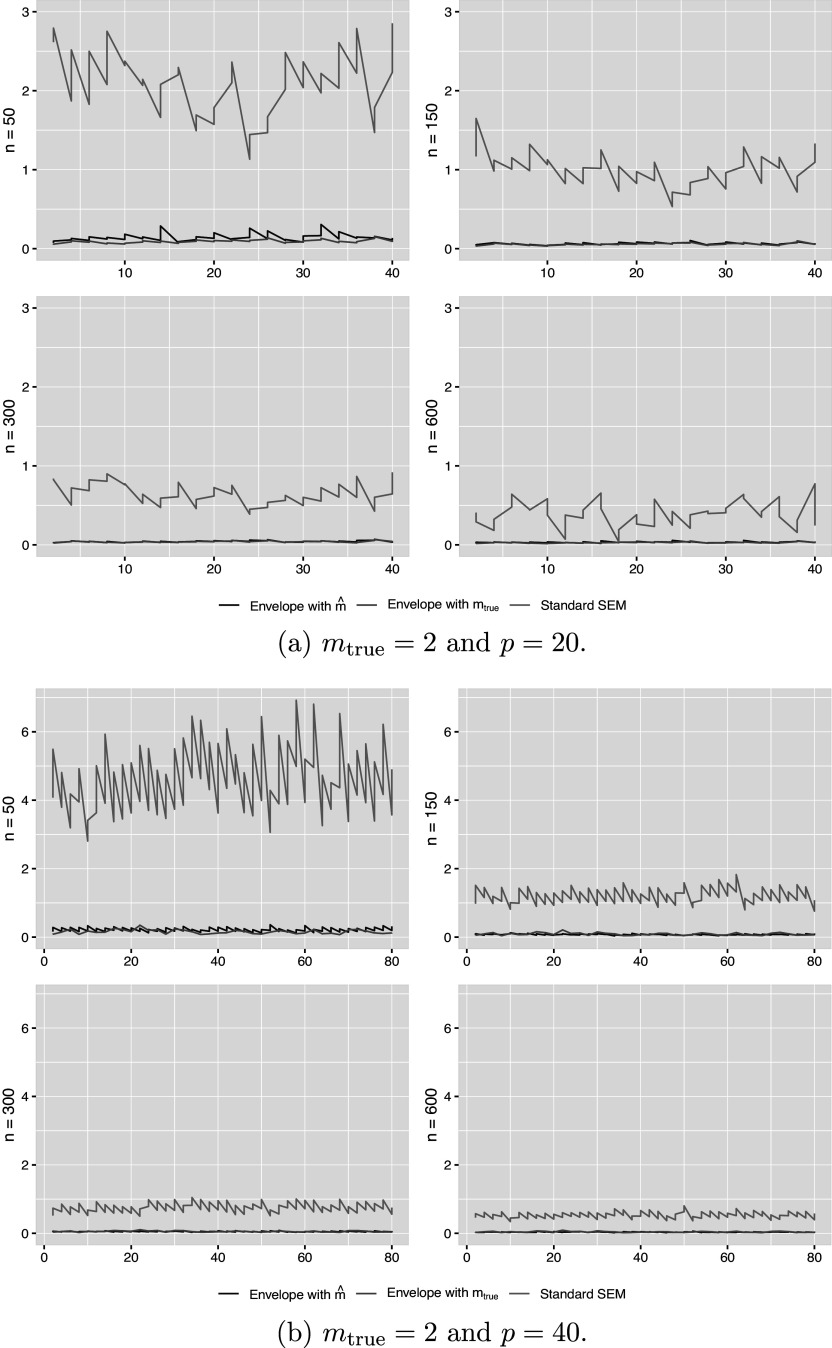
The RMSE of the estimated elements of 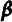 for two cases in simulation. *Note*: x-axis: coordinate of 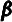 . y-axis: value of RMSE.

Figure 1 and Tables 2 and 3 display the root mean squared error (RMSE) and bias of the estimated 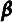 for two specific settings of 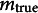 and 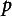 . The plot includes the selected model with 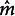 , the true model with 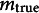 , and the standard SEM for two settings of 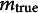 and 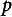 . The explicit values of the RMSE and bias of several selected elements in 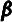 are presented in Tables 2 and 3, respectively. The figure and the tables demonstrate that the envelope model can achieve efficiency gains and significantly reduce the RMSE and bias compared to the standard SEM. This improvement is particularly pronounced when the sample size is small. The performance for the other settings is similar, and the figures are not presented. Increasing the number of replications, e.g., to 200, only slightly reduced the RMSE and bias of the 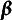 estimates further. Figure S2 in the Supplementary Material provides detailed variations observed in the estimated 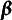 elements under the scenario of 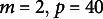 to illustrate this marginal improvement from additional replications. Moreover, as the sample size increases, the selected model in the envelope model tends to behave similarly to the true envelope model, and their performance in terms of RMSE becomes almost indistinguishable. Regarding the estimation of the latent factor matrix, Table 4 shows that all three methods perform well with small RMSE values, and there is no noticeable difference among them. This suggests that the SEM can incorporate the envelope structure in the structural equation without compromising the accuracy of the measurement equation. The results for the other settings exhibit similar performance and thus are not reported.

The enhanced estimation accuracy of BESEM gained from the underlying envelope space is further corroborated through a comparison with SEM utilizing BLasso, a widely employed regularized SEM approach (Feng et al., 2015). Table S1 and Figure S3 in the Supplementary Material present the bias and RMSE of the 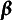 coefficients estimated by the proposed BESEM, standard SEM, and SEM with BLasso. Notably, BESEM exhibits minimal bias and RMSE, standard SEM shows the highest bias and RMSE, whereas SEM with BLasso falls in between.

We conduct several additional simulations to evaluate further the robustness of the proposed method in dimension selection and parameter estimation. The first one considers the scenario, where the lower-dimensional envelope subspace is non-existent, i.e., 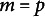 . The second one examines the validity of the proposed method in terms of violating the normality assumptions on the distribution of predictors by generating 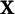 from heavy-tailed and skewed distributions. Additionally, we perform prior sensitivity analyses with respect to different choices of hyperparameters. Performance of the proposed method is stable across the scenarios. Detailed setups and results are provided in the Supplementary Material.

In summary, the envelope model, including the selected model and the true model, outperforms the standard SEM model, especially in estimating 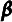 . While information criteria may exhibit limitations in terms of selection rate, it is noteworthy that as the sample size increases, the selected model within the envelope model framework can approximate the true model closely. This observation highlights the feasibility of the AWE method in selecting appropriate envelope models and maintaining their effectiveness in estimating the parameter of interest efficiently.

**Table 2:** The RMSE of estimated elements in 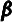 for two cases in Simulation

(a) 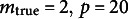												
Method	Envelope 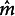				Envelope 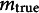				SEM			
*n*	50	150	300	600	50	150	300	600	50	150	300	600
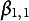	0.0952	0.0494	0.0269	0.0226	0.0568	0.0326	0.0224	0.0166	2.7929	1.6482	0.8245	0.2910
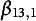	0.2243	0.1023	0.0650	0.0419	0.1361	0.0817	0.0591	0.0492	1.6694	0.8369	0.5374	0.3805
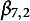	0.1040	0.0497	0.0309	0.0212	0.0777	0.0460	0.0312	0.0217	1.6624	0.8231	0.4722	0.3380
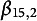	0.0826	0.0685	0.0345	0.0256	0.0843	0.0558	0.0403	0.0241	2.0392	0.7557	0.4977	0.4065

*Note*: Displayed are several randomly selected elements in 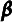 .

**Table 3:** The bias of estimated elements in 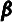 for two cases in simulation

(a) 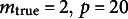												
Method	Envelope 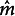				Envelope 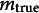				SEM			
*n*	50	150	300	600	50	150	300	600	50	150	300	600
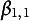	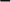 0.0038	0.0025	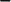 0.0025	0.0047	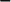 0.0084	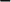 0.0073	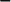 0.0004	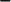 0.0035	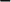 0.0069	0.0887	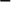 0.0571	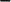 0.2859
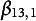	0.0196	0.0035	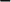 0.0101	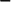 0.0001	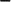 0.0083	0.0095	0.0004	0.0045	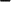 0.1245	0.1159	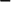 0.0516	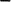 0.0472
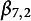	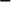 0.0054	0.0000	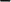 0.0036	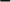 0.0022	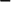 0.0022	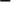 0.0087	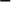 0.0038	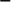 0.0029	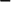 0.0516	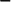 0.0532	0.0351	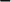 0.3362
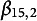	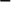 0.0045	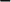 0.0041	0.0058	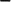 0.0005	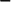 0.0006	0.0032	0.0024	0.0018	0.2290	0.0346	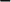 0.0659	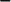 0.4060

*Note*: Displayed are several randomly selected elements in 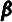 .

**Table 4:** The RMSE of estimated free elements in 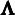 for two cases in simulation

(a) 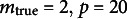												
Method	Envelope 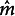				Envelope 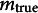				SEM			
*n*	50	150	300	600	50	150	300	600	50	150	300	600
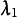	0.0014	0.0008	0.0006	0.0003	0.0015	0.0007	0.0006	0.0004	0.0013	0.0007	0.0006	0.0008
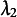	0.0038	0.0019	0.0015	0.0011	0.0039	0.0021	0.0015	0.0010	0.0038	0.0021	0.0015	0.0014
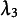	0.0044	0.0030	0.0017	0.0014	0.0047	0.0028	0.0019	0.0014	0.0051	0.0028	0.0018	0.0012
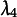	0.0041	0.0025	0.0015	0.0013	0.0044	0.0023	0.0017	0.0013	0.0047	0.0022	0.0015	0.0007

To obtain the results reported in Table 1 using the proposed method, the computational time per replication ranges from about 1 min to a maximum of approximately 17 min on a Linux machine running R with a CPU block speed of 2.60 GHz. The actual timing depends on the specific combination of sample size, predictor dimension, and envelope dimension. Detailed results are summarized in Table S2 in the Supplementary Material. The code for implementing the preceding analysis is scripted in R with Rcpp and is freely available in the Supplementary Material.

## 5 Real data analysis

We apply the proposed BESEM to analyze the ADNI dataset. The ADNI project, launched in 2004, aims to advance research on the early detection, diagnosis, tracking, and treatment of AD and other forms of cognitive impairment. It has become a widely recognized and extensively studied collection of neuroimaging, clinical, and biomarker data related to AD. One crucial topic in the study of AD and other dementia diseases is understanding the relationship between cognitive decline and changes in brain structure. Previous studies have indicated that various brain regions are affected to different extents in AD (Bartos et al., 2019; Jones et al., 2006). In our study, we integrate neuroimaging phenotypes comprising volumetric and cortical thickness measures derived from MRI data. These measures capture the structural characteristics of the brain across a moderate number of ROIs. We incorporate these neuroimaging phenotypes into a regression model and utilize the envelope technique to gain new insights into the relationship between brain structure and cognitive ability.

Clinical diagnostic tools, including neurological exams and cognitive and functional assessments, are believed to reflect the degree of cognitive impairment and monitor disease progression (Albert et al., 2013). Therefore, we regard cognitive impairment as a latent variable denoted by 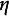 . We consider five different test scores from the ADNI study to assess cognitive impairment in various domains. The first test score is the Mini-Mental State Examination (MMSE), a widely used screening tool that assesses cognitive impairment from various cognitive domains, including orientation, attention and calculation, recall, language, and visuospatial abilities. The second test score is the AD Assessment Scale-Cognitive subscale 13 (ADAS13), which is specifically designed for use in clinical trials to measure cognitive changes associated with AD. The scoring of ADAS13 is more complex than that of MMSE. Higher ADAS13 scores indicate severe cognitive impairment. The third one is the Functional Assessment Questionnaire (FAQ), which evaluates an individual’s functional ability to perform activities of daily living such as dressing, bathing, grooming, eating, and managing finances. The final two test scores are derived from the Rey Auditory Verbal Learning Test (RAVLT), a neuropsychological test that assesses verbal learning and memory. Specifically, we selected two measures from RAVLT: RAVLT Immediate (RAVLT.immediate) and RAVLT percent forgetting (RAVLT.perc.forgetting). These two measures are selected since they capture different aspects of episodic memory and have been shown to be closely related to AD detection by previous research (Estévez-González et al., 2003; Moradi et al., 2017). To ensure consistency in interpreting the test scores, we reversed the MMSE and RAVLT.immediate scores so that higher values indicate a severe impairment in cognitive function. Additionally, all five test scores were standardized before conducting the analysis. These standardized test scores are denoted as 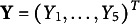 , as the response variables in equation (8).

The MRI brain imaging data were preprocessed by UCSF using Freesurfer methods, which involved segmenting the T1 weighted images into small regions to define volumetric and cortical thickness values. Detailed parcellation and quality control (QC) guidelines can be found on the LONI-ADNI website. We followed the instructions provided by Szefer et al. (2017), and Greenlaw et al. (2017) and include 56 derived neroimaging phenotypes. A description of these phenotypes can be found in Table 5. The log-transformed values of the volumetric or cortical thickness measures serve as the predictors 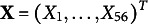 in the structural envelope model.

**Table 5:** Imaging phenotypes defined as volumetric or cortical thickness measures of 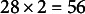 ROIs

ID	Measurement	ROI
AmygVol	Volume	Amygdala
CerebCtx	Volume	Cerebral cortex
CerebWM	Volume	Cerebral white matter
HippVol	Volume	Hippocampus
InfLatVent	Volume	Inferior lateral ventricle
LatVent	Volume	Lateral ventricle
EntCtx	Thickness	Entorhinal cortex
Fusiform	Thickness	Fusiform gyrus
InfParietal	Thickness	Inferior parietal gyrus
InfTemporal	Thickness	Inferior temporal gyrus
MidTemporal	Thickness	Middle temporal gyrus
Parahipp	Thickness	Parahippocampal gyrus
PostCing	Thickness	Posterior cingulate
Postcentral	Thickness	Postcentral gyrus
Precentral	Thickness	Precentral gyurs
Precuneus	Thickness	Precuneus
SupFrontal	Thickness	Superior frontal gyrus
SupParietal	Thickness	Superior parietal gyrus
SupTemporal	Thickness	Superior temporal gyrus
Supramarg	Thickness	Supramarginal gyrus
TemporalPole	Thickness	Temporal pole
MeanCing	Mean thickness	Caudal anterior cingulate, isthmus cingulate, posterior cingulate, rostral anterior cingulate
MeanFront	Mean thickness	Caudal midfrontal, rostral midfrontal, superior frontal, lateral orbitofrontal, and medial orbitofrontal gyri, frontal pole
MeanLatTemp	Mean thickness	Inferior temporal, middle temporal, and superior temporal gyri
MeanMedTemp	Mean thickness	Fusiform, parahippocampal, and lingual gyri, temporal pole and transverse temporal pole
MeanPar	Mean thickness	Inferior and superior parietal gyri, supramarginal gyrus, and precuneus
MeanSensMotor	Mean thickness	Precentral and postcentral gyri
MeanTemp	Mean thickness	Inferior temporal, middle temporal, superior temporal, fusiform, parahippocampal, lingual gyri, temporal pole, transverse temporal pole

*Note*: Each phenotype in the table corresponds to the two phenotypes for the left and right hemispheres.

The original dataset from the ADNI-1 phase comprises clinical and imaging measurements from 800 participants, with missing data in both cognitive test scores and brain imaging data. After excluding missing data, a total of 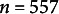 observations are retained in the analysis. Among these observations, 169 individuals are diagnosed with cognitive normal (CN), 271 have mild cognitive impairment (MCI), and 117 are diagnosed with AD. We apply the proposed BESEM method to this dataset, using a flat prior similar to that described in Section 4.

The MCMC algorithm converges within the initial thousands of iterations, as shown by the trace plots depicted in Figure S4 in the Supplementary Material. Therefore, a total of 20000 samples are drawn from the conditional posterior density, with the first 8000 iterations discarded as the burn-in stage. The AIC, BIC, DIC, and AWE methods were used to determine the optimal dimension of the envelope structure. All these methods consistently indicate that the optimal dimension is 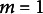 . Therefore, the subsequent estimation results using the BESEM method are based on the envelope model with a dimension of 1.

**Figure 2:**
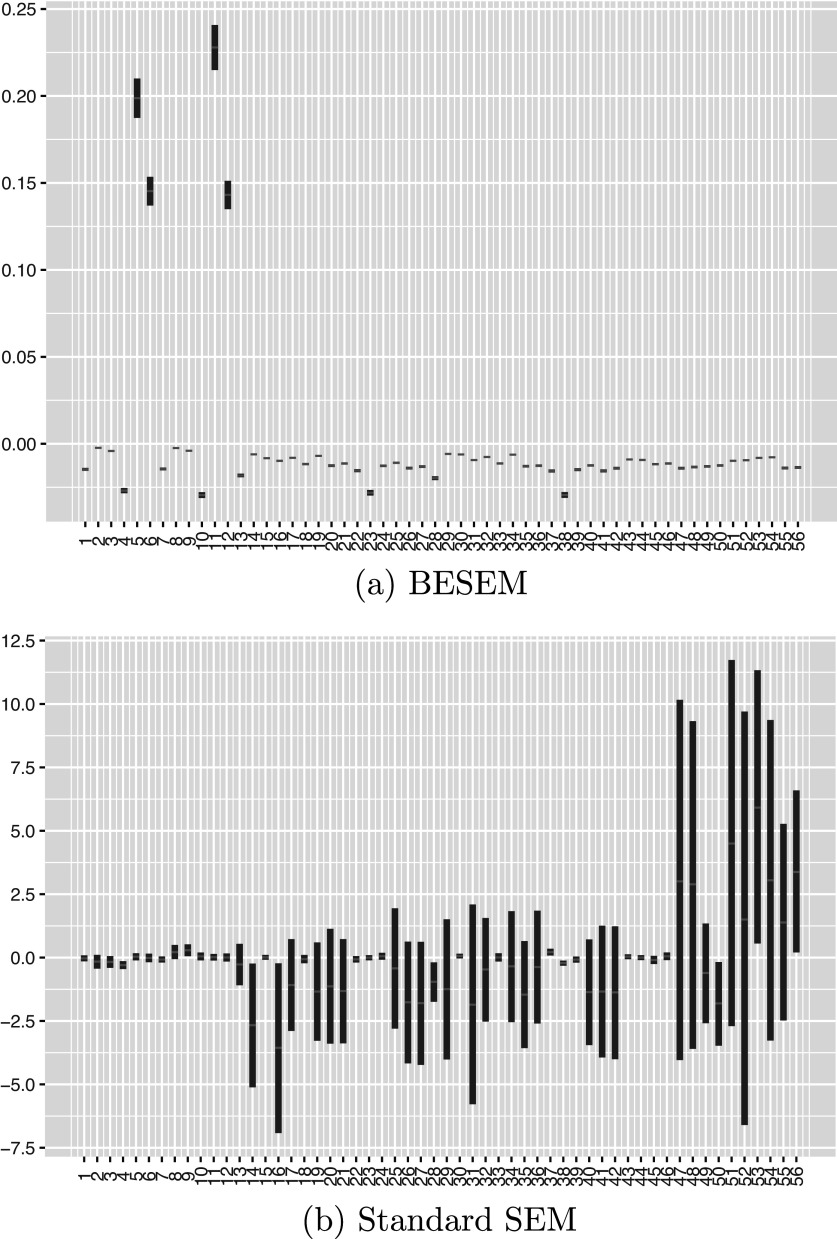
Point and 95% interval estimates of each element of 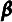 for the ADNI study. *Note*: x-axis: ID of each ROI, which aligns with the order in Table 5 (adjacent numbers represent the left hemisphere and right hemisphere, respectively). y-axis: Estimated value. Red short line: the value of the estimated coefficient. Grey rectangle: 95% CI.

The point estimate of the factor loading 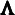 is 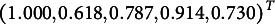 , with a negligible standard error estimate for each element. The estimates of coefficients 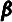 (Est), along with their standard error estimates (SE), and 95% credible interval (CI) of the posterior samples, are displayed in Figure 2a and Table 6. Notably, the inferior lateral ventricle and lateral ventricle for both hemispheres are found to have a significant effect under the envelope structure, while the remaining ROIs exhibit minimal impact, with estimated coefficients close to zero. This finding aligns with previous research by Bartos et al. (2019), which suggests that the inferior regions of both lateral ventricles have the most significant enlargements in individuals with AD.

**Table 6:**
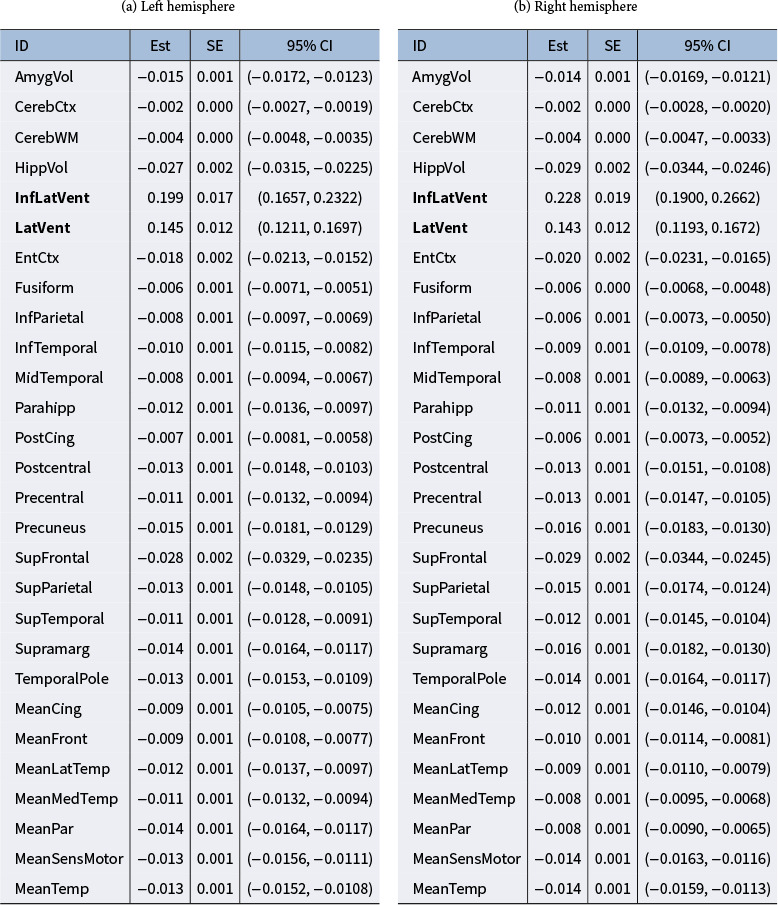
Point estimates (Est), Standard Error Estimates (SE), and 95% CIs of the elements in 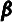 by BESEM method in ADNI study

For comparison, we also present the results obtained using the standard SEM method in Figure 2b and Table 7. Akin to the simulation study, the standard SEM was estimated using a Bayesian approach on the subset of 557 subjects with complete measurements, ensuring a fair comparison with the proposed BESEM. In line with standard Bayesian SEM practices, we assigned conjugate normal-inverse-gamma priors to parameters in the measurement equation and vague normal-inverse-Wishart priors to parameters in the structural equation to derive Bayesian estimates. These results demonstrate a conspicuous deviation from those obtained using the envelope method. In this analysis, we exclude regions with point estimates close to zero and those with 95% CIs that include zero and identify several ROIs that exhibited significance in the regression model. The selected regions are marked with bold text in Table 7. The selected significant regions do not exhibit consistency between the two brain hemispheres. The fusiform and inferior temporal in the left hemisphere is shown to be more significant, which aligns with the notion proposed by Galton et al. (2001). Meanwhile, we also found a significant impact on the hippocampus volume in the left hemisphere but not in the right hemisphere. This finding contradicts the conclusion in Barnes et al. (2005) and Yang et al. (2017), which suggests a preferential association of the right hippocampus with AD. Such partial inconsistency with the existing literature is likely a consequence of multicollinearity among the covariates and overfitting to the noise or the immaterial information in the multivariate regression problem, thereby raises doubts about the validity of the results. It demonstrates that the standard SEM model may yield results lacking interpretability or mask some significant associations in the setting of complex covariates that can be projected to a reducing subspace. The estimation results of SEM with BLasso are also presented in Table S3 and Figure S5 in the Supplementary Material for a comprehensive comparison. A similar pattern to the standard SEM is observed. Moreover, in this dataset, the changes observed in different ROIs are not entirely independent, and a plain linear regression model may not adequately capture the interdependencies among the covariates in such cases, further contributing to noisy and potentially misleading results. In contrast, the BESEM method enhances the identification of important predictors and significantly improves estimation efficiency. It better handles the challenges posed by high-dimensional covariates and accounts for the interdependencies among ROIs, leading to more reliable and informative results.

**Table 7:**
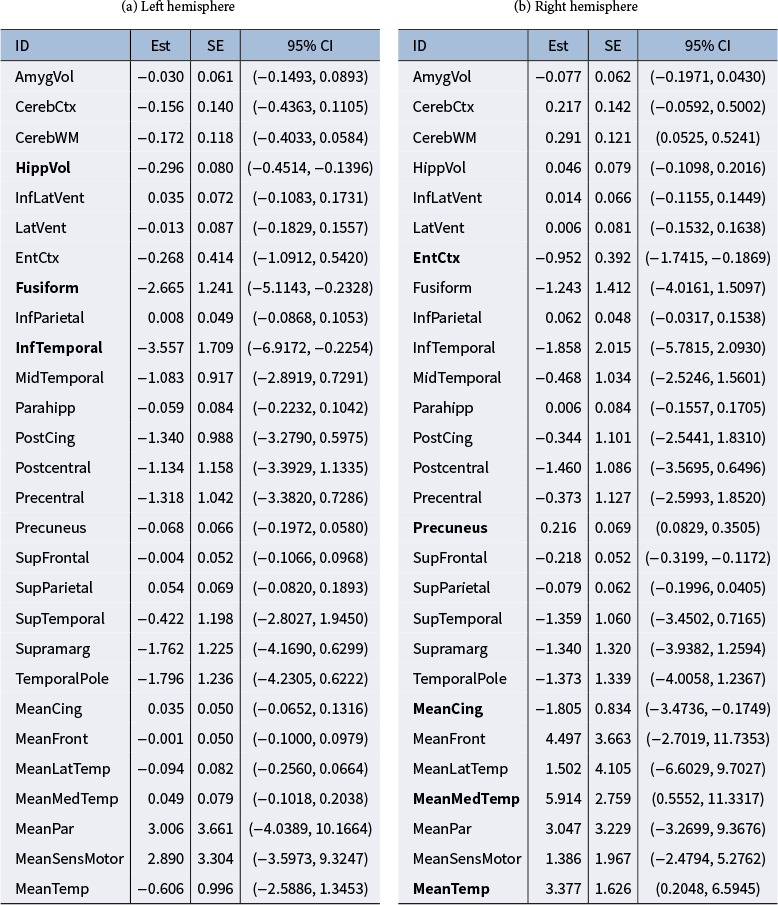
Point estimates (Est), Standard Error Estimates (SE), and 95% CI of the elements in 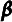 by standard SEM method in ADNI study

## 6 Conclusion and discussion

This article introduces a BESEM that incorporates a predictor envelope structure in the structural equation, in conjunction with a factor model, to achieve dimension reduction. The response variables are grouped into latent factors with a predetermined structure, allowing for dimension reduction of multiple responses in the factor model. In the structural equation, the concerned predictors are decomposed into two parts, the material part and the immaterial part. These two parts are independent, with the immaterial part containing no information relevant to the latent factor. The presence of the envelope structure, along with a small envelope dimension, can greatly reduce the number of unknown parameters, leading to improved estimation efficiency. The application of the proposed model to the ADNI dataset demonstrates its potential in identifying important covariates.

While there are still several areas of interest that warrant further investigation. First, there is a need to develop an efficient method for selecting the envelope dimension. Currently, we approach this as a model selection task and utilize information criteria to identify the optimal envelope structure, which can lead to overestimated envelope dimension. A potential solution to mitigate this limitation involves circumventing the selection of envelope dimension by developing a weighted average variant of the envelope estimator, using the information criterion values as potential weights (Eck & Cook, 2017). Nonetheless, this approach may introduce theoretical and computational challenges, and we consider it as a promising future direction that warrants continuing effort. Besides, employing information criteria for envelope dimension can be time-consuming and computationally intensive. To address this, Zhang & Mai (2018) proposed two unified approaches, full Grassmannian (FG) optimization and 1 dimension (1D) selection, which provide theoretical justifications for selection consistency and exhibit computational stability. Adapting this idea from a Bayesian perspective holds promise, and we aim to develop a method that can simultaneously select the dimension and perform estimation. Second, the substitution of the CFA model with an EFA model is worth exploring. This would relax the assumption of a predetermined number of latent factors and instead allow the factor loading matrix to be determined by both prior information and data. However, determining the number of latent factors can be challenging, especially when it is linked to predictors with an uncertain envelope structure. Third, intricate missing mechanisms can arise in multivariate regression while addressing missing data within envelope models is an ongoing area of research with few established methodologies. We see the accommodation of diverse missing mechanisms in the joint modeling of envelope and SEM as a potential for further exploration. Lastly, it is worth considering the possibility of incorporating the envelope structure into the multivariate variables (responses) in the measurement component of an SEM. Cook & Zhang (2015b) introduced envelopes that simultaneously reduce predictors and responses in a multivariate linear regression model, leading to significant improvements over traditional methods. Therefore, we consider it a promising future direction to further extend the BESEM to incorporate an envelope structure for the multivariate responses in the measurement component. For instance, if we can appropriately separate the immaterial part of the response variables in the measurement equation and group the material part into the latent factors, then it is possible to handle high-dimensional response vectors in BESEM. Substantial efforts are required to achieve this advancement.

## Supporting information

Sun et al. supplementary material Sun et al. supplementary material

## Supplementary material

The supplementary material for this article can be found at https://doi.org/10.1017/psy.2025.10027.
