# Supplementary material for: Bayesian Structural Equation Envelope Model
Source: Psychometrika. 2025 Aug 8;90(4):1236–57. doi: 10.1017/psy.2025.10027 (PMC12660024; doi:10.1017/psy.2025.10027)
Supplement: Sun et al. supplementary material [file S0033312325100276sup001.pdf]

Supplementary Material for "Bayesian Structural Equation Envelope Model"

S.1 MCMC Algorithm

Algorithm 1. One iteration of the Metropolis-within-Gibbs sampler in BESEM with envelope dimension $0 < m < p$.

Step 1: Draw samples of $\mathbf{\Lambda}_{yk}$ and $\sigma^2_{\varepsilon k}$ for $k = 1, \ldots, p$:

Let λ_{ykj} be the (k, j)-th elements of $\mathbf{\Lambda}_y$ for $j = 1, \ldots, 1 + q$, and $k = 1, \ldots, p$, and $\mathbf{u}_i = (\mathbf{1}_p^T, \boldsymbol{\eta}_i^T)^T$. To specify the position of the fixed elements in $\mathbf{\Lambda}_y$, we introduce an index matrix $\mathbf{L}_y$ with the elements $l_{ykj} = 0$ if λ_{ykj} if fixed, otherwise $l_{ykj} = 1$, for $j = 1, \ldots, 1 + q$, and $k = 1, \ldots, p$.

Let $\mathbf{U} = (\mathbf{u}_1, \ldots, \mathbf{u}_n)$ and $\mathbf{U}_k$ be the submatrix of $\mathbf{U}$ such that all he rows corresponding to $l_{ykj} = 0$ is deleted, and let $\mathbf{Y}_k^{*T} = (y^*_{1k}, \ldots, y^*_{nk})$ with

$$y^*_{ik} = y_{ik} - \sum_{j=1}^{1+q} \lambda_{ykj} u_{ij} (1 - l_{ykj}),$$

where u_{ij} is the (i, j)-th element of $\mathbf{U}$. Then we draw samples of $\mathbf{\Lambda}_{yk}$ and $\sigma^2_{\varepsilon k}$ from their posterior distributions:

$$\left[\mathbf{\Lambda}_{yk} \mid \mathbf{Y}, \boldsymbol{\eta}, \sigma^2_{\varepsilon k}\right] \sim \mathrm{N}\left(\boldsymbol{\mu}_{\mathbf{\Lambda} yk}, \sigma^2_{\varepsilon k} \mathbf{\Sigma}^{-1}_{\mathbf{\Lambda} k}\right), \qquad \left[\sigma^2_{\varepsilon k} \mid \mathbf{Y}, \boldsymbol{\eta}\right] \sim \mathrm{IG}\left(\frac{n}{2} + a_{0k}, b^*_k\right),$$

where $\boldsymbol{\mu}_{\mathbf{\Lambda} yk} = \mathbf{\Sigma}_{\mathbf{\Lambda} k}\left(\mathbf{H}^{-1}_{0k}\mathbf{\Lambda}_{0yk} + \mathbf{U}_k \mathbf{Y}^*_k\right)$, $\mathbf{\Sigma}_{\mathbf{\Lambda} k} = \left(\mathbf{H}^{-1}_{0y} + \mathbf{U}_k \mathbf{U}_k^T\right)^{-1}$, and

$$b^*_k = b_{0k} + \frac{1}{2}\left(\mathbf{Y}_k^{*T}\mathbf{Y}^*_k - \boldsymbol{\mu}^T_{\mathbf{\Lambda} yk}\mathbf{\Sigma}^{-1}_{\mathbf{\Lambda} k}\boldsymbol{\mu}_{\mathbf{\Lambda} yk} + \mathbf{\Lambda}^T_{0yk}\mathbf{H}^{-1}_{0k}\mathbf{\Lambda}_{0yk}\right).$$

Step 2: Generate $\left(\boldsymbol{\mu}^T_{\mathbf{X}}, \boldsymbol{\mu}^T_{\eta}\right)^T$ from $N(\bar{\boldsymbol{D}}, \frac{1}{n}\mathbf{\Sigma}_{\boldsymbol{D}})$, where

$$\bar{\boldsymbol{D}} = \begin{bmatrix} \bar{\mathbf{X}} \\ \bar{\boldsymbol{\eta}} \end{bmatrix}, \qquad \mathbf{\Sigma}_{\boldsymbol{D}} = \begin{bmatrix} \mathbf{\Phi}(\boldsymbol{A})\mathbf{\Delta}\mathbf{\Phi}(\boldsymbol{A})^T + \mathbf{\Phi}_0(\boldsymbol{A})\mathbf{\Delta}_0\mathbf{\Phi}_0(\boldsymbol{A})^T & \mathbf{\Phi}(\boldsymbol{A})\mathbf{\Delta}\boldsymbol{c} \\ \boldsymbol{c}^T\mathbf{\Delta}\mathbf{\Phi}^T & \mathbf{\Sigma}_\delta + \boldsymbol{c}^T\mathbf{\Delta}\boldsymbol{c} \end{bmatrix},$$

Step 3: Generate $\boldsymbol{c}$ from $\mathcal{MN}_{m,q}\left(\mathbf{M}_1^{-1}\boldsymbol{\Phi}(\boldsymbol{A})^T\boldsymbol{e}_1, \mathbf{M}_1^{-1}, \boldsymbol{\Sigma}_\delta\right)$, where $\mathbf{M}_1 = \mathbf{M}+\boldsymbol{\Phi}(\boldsymbol{A})^T\mathbb{X}_{\boldsymbol{\mu}_{\mathbf{X}}}^T\boldsymbol{\eta}_{\boldsymbol{\mu}_{\boldsymbol{\eta}}}\boldsymbol{\Phi}(\boldsymbol{A})$, $\boldsymbol{e}_1 = \boldsymbol{e} + \mathbb{X}_{\boldsymbol{\mu}_{\mathbf{X}}}^T\boldsymbol{\eta}_{\boldsymbol{\mu}_{\boldsymbol{\eta}}}$, $\mathbb{X}_{\boldsymbol{\mu}_{\mathbf{X}}} = \mathbb{X} - \mathbf{1}_n\boldsymbol{\mu}_{\mathbf{X}}^T$ and $\boldsymbol{\eta}_{\boldsymbol{\mu}_{\boldsymbol{\eta}}} = \boldsymbol{\eta} - \mathbf{1}_n\boldsymbol{\mu}_\eta^T$.

Step 4: Generate $\boldsymbol{\Sigma}_\delta$ from $\mathrm{IW}_q\left(\tilde{\boldsymbol{\Psi}}_\delta, \tilde{v}_\delta\right)$, where $\tilde{v}_\delta = v_0 + n + m$, and

$$\tilde{\boldsymbol{\Psi}}_\delta = \boldsymbol{\Psi}_0 + \left(\boldsymbol{\eta}_{\boldsymbol{\mu}_{\boldsymbol{\eta}}} - \mathbb{X}_{\boldsymbol{\mu}_{\mathbf{X}}}\boldsymbol{\Phi}(\boldsymbol{A})\boldsymbol{c}\right)^T\left(\boldsymbol{\eta}_{\boldsymbol{\mu}_{\boldsymbol{\eta}}} - \mathbb{X}_{\boldsymbol{\mu}_{\mathbf{X}}}\boldsymbol{\Phi}(\boldsymbol{A})\boldsymbol{c}\right) + \left(\boldsymbol{c} - \mathbf{M}^{-1}\boldsymbol{\Phi}(\boldsymbol{A})^T\boldsymbol{e}\right)^T\mathbf{M}\left(\boldsymbol{c} - \mathbf{M}^{-1}\boldsymbol{\Phi}(\boldsymbol{A})^T\boldsymbol{e}\right).$$

Step 5: Generate $\boldsymbol{\Delta}$ from $\mathrm{IW}_m\left(\boldsymbol{\Psi}_1 + \boldsymbol{\Phi}(\boldsymbol{A})^T\mathbb{X}_{\boldsymbol{\mu}_{\mathbf{X}}}^T\mathbb{X}_{\boldsymbol{\mu}_{\mathbf{X}}}\boldsymbol{\Phi}(\boldsymbol{A}), v_1 + n\right)$.

Step 6: Generate $\boldsymbol{\Delta}_0$ from $\mathrm{IW}_{p-m}\left(\boldsymbol{\Psi}_2 + \boldsymbol{\Phi}_0(\boldsymbol{A})^T\mathbb{X}_{\boldsymbol{\mu}_{\mathbf{X}}}^T\mathbb{X}_{\boldsymbol{\mu}_{\mathbf{X}}}\boldsymbol{\Phi}_0(\boldsymbol{A}), v_1 + n\right)$.

Step 7: Generate $\boldsymbol{A}$ with Metropolis sampler:

The posterior density of $\boldsymbol{A}$ is:

$$p(\boldsymbol{A} \mid \boldsymbol{\eta}, \boldsymbol{c}, \boldsymbol{A}, \mathbb{X}) \propto p(\boldsymbol{\eta} \mid \boldsymbol{\mu}_{\boldsymbol{\eta}}, \boldsymbol{\mu}_{\mathbf{X}}, \boldsymbol{\Sigma}_\delta, \boldsymbol{c}, \boldsymbol{A}, \mathbb{X})p(\mathbf{X} \mid \boldsymbol{\mu}_{\mathbf{X}}, \boldsymbol{\Sigma}_{\mathbf{X}})p(\boldsymbol{c} \mid \boldsymbol{A}, \boldsymbol{\Sigma}_\delta)p(\boldsymbol{A}),$$

We let $h(\boldsymbol{A})$ denote the log density of $[\boldsymbol{A} \mid \boldsymbol{\eta}, \boldsymbol{c}, \boldsymbol{A}, \mathbb{X}]$, then we have

It is clear that the posterior distribution does not have a closed form, so we use the Metropolis-Hastings algorithm to draw samples for $\boldsymbol{A}$. Let $\boldsymbol{a}_j \in \mathbb{R}^{p-m}$ represent the j-th column of $\boldsymbol{A}$ for, $j = 1, \ldots, m$, we update $\boldsymbol{A}$ column by column. The proposal distribution for each $\boldsymbol{a}_j$ is $N_{p-m}(\boldsymbol{a}_j, \tau\mathbf{I}_{p-m})$, where τ is the tuning parameter that can be adjusted to control the acceptance rate.

By iteratively updating each column of $\boldsymbol{A}$ using the Metropolis-Hastings algorithm, we can generate a sequence of samples from the posterior distribution of $\boldsymbol{A}$. The tuning parameter τ can be adjusted to achieve an acceptance rate of around 70%, which can achieve an efficient sampling.

Step 8: Generate $\boldsymbol{\eta}_i, i = 1, \ldots, n$, from $\mathrm{N}_q\left(\mu^*_{\boldsymbol{\eta}_i}, \boldsymbol{\Sigma}^*_{\boldsymbol{\eta}_i}\right)$, where

$$\mu^*_{\boldsymbol{\eta}_i} = \boldsymbol{\Sigma}^*_{\boldsymbol{\eta}_i}\left[\boldsymbol{\Lambda}_y^T\boldsymbol{\Sigma}_\varepsilon^{-1}(\mathbf{Y}_i - \boldsymbol{\mu}_{\mathbf{Y}}) + \boldsymbol{\Sigma}_\delta^{-1}\left(\boldsymbol{\mu}_{\boldsymbol{\eta}} + \boldsymbol{c}^T\boldsymbol{\Phi}(\boldsymbol{A})^T(\mathbf{X}_i - \boldsymbol{\mu}_{\mathbf{X}})\right)\right],$$
$$\boldsymbol{\Sigma}^*_{\boldsymbol{\eta}_i} = \left[\boldsymbol{\Lambda}_y^T\boldsymbol{\Sigma}_\varepsilon^{-1}\boldsymbol{\Lambda}_y + \boldsymbol{\Sigma}_\delta^{-1}\right]^{-1}.$$

For the cases $m = 0$ and $m = p$, Algorithm 1 can be modified by discarding the steps that draw samples of $\boldsymbol{A}$. In addition, when $m = 0$, then $\boldsymbol{c} = \mathbf{0}$ and $\boldsymbol{\Phi}_0(\boldsymbol{A}) = \mathbf{I}_p$, $\boldsymbol{\Sigma}_{\mathbf{X}} = \boldsymbol{\Delta}_0$, and Step 3. and Step 6. are not needed.

S.2 Theoretical Proofs

S.2.1 Proof of Theorem 1

Proof. The objective is to show the integrability of the posterior density $p(\boldsymbol{\theta}, \boldsymbol{\eta} \mid \mathcal{D})$ with respect to Lebesgue measure on $\mathbb{R}^r \times \mathbb{R}^{r-q} \times \mathbb{R}^r_+ \times \mathbb{S}^{q\times q}_+ \times \mathbb{R}^q \times \mathbb{R}^p \times \mathbb{S}^{m\times m}_+ \times \mathbb{S}^{(p-m)\times(p-m)}_+ \times \mathbb{R}^{m\times q} \times \mathbb{R}^{(p-m)\times m} \times \mathbb{R}^q$. Due to the proper prior of $\{\boldsymbol{\mu}_{\mathbf{Y}}, \boldsymbol{\Lambda}, \boldsymbol{\Sigma}_\varepsilon\}$, it is sufficient to show

$$\int p(\boldsymbol{\Sigma}_\delta, \boldsymbol{\mu}_\eta, \boldsymbol{\mu}_{\mathbf{X}}, \boldsymbol{\Delta}, \boldsymbol{\Delta}_0, \boldsymbol{c}, \boldsymbol{A}, \boldsymbol{\eta} \mid \mathcal{D}) d\tilde{\boldsymbol{\theta}}\boldsymbol{\eta} < \infty. \tag{1}$$

Let $\tilde{\boldsymbol{\theta}} = (\boldsymbol{\Sigma}_\delta, \boldsymbol{\mu}_\eta, \boldsymbol{\mu}_{\mathbf{X}}, \boldsymbol{\Delta}, \boldsymbol{\Delta}_0, \boldsymbol{c}, \boldsymbol{A})$. Since $p(\tilde{\boldsymbol{\theta}}, \boldsymbol{\eta} \mid \mathcal{D}) \propto p(\mathcal{D}, \boldsymbol{\eta} \mid \tilde{\boldsymbol{\theta}})p(\tilde{\boldsymbol{\theta}})$ we have

$$\begin{aligned}
&\log p(\tilde{\boldsymbol{\theta}}, \boldsymbol{\eta} \mid \mathcal{D}) \\
= &-\frac{n}{2}\log|\boldsymbol{\Sigma}_\delta| - \frac{1}{2}\mathrm{tr}\left[\left(\boldsymbol{\eta} - \mathbf{1}_n\boldsymbol{\mu}_{\boldsymbol{\eta}}^T - \mathbb{X}_{\boldsymbol{\mu}_{\mathbf{X}}}\boldsymbol{\Phi}(\boldsymbol{A})\boldsymbol{c}\right)\boldsymbol{\Sigma}_\delta^{-1}\left(\boldsymbol{\eta} - \mathbf{1}_n\boldsymbol{\mu}_{\boldsymbol{\eta}}^T - \mathbb{X}_{\boldsymbol{\mu}_{\mathbf{X}}}\boldsymbol{\Phi}(\boldsymbol{A})\boldsymbol{c}\right)^T\right] \\
&-\frac{n}{2}\log|\boldsymbol{\Delta}| - \frac{n}{2}\log|\boldsymbol{\Delta}_0| - \frac{1}{2}\mathrm{tr}\left[\mathbb{X}_{\boldsymbol{\mu}_{\mathbf{X}}}\boldsymbol{\Sigma}_\delta^{-1}\mathbb{X}_{\boldsymbol{\mu}_{\mathbf{X}}}^T\right] \\
&-\frac{m}{2}\log|\boldsymbol{\Sigma}_\delta| - \frac{1}{2}\mathrm{tr}\left[\boldsymbol{\Sigma}_\delta^{-1}\left(\boldsymbol{c} - \boldsymbol{M}^{-1}\boldsymbol{\Phi}(\boldsymbol{A})^T\boldsymbol{e}\right)^T\boldsymbol{M}\left(\boldsymbol{c} - \boldsymbol{M}^{-1}\boldsymbol{\Phi}(\boldsymbol{A})^T\boldsymbol{e}\right)\right] \\
&-\frac{v_1 + m + 1}{2}\log|\boldsymbol{\Delta}| - \frac{1}{2}\mathrm{tr}\left[\boldsymbol{\Delta}^{-1}\boldsymbol{\Psi}_1\right] - \frac{v_2 + p - m + 1}{2}\log|\boldsymbol{\Delta}_0| - \frac{1}{2}\mathrm{tr}\left[\boldsymbol{\Delta}_0^{-1}\boldsymbol{\Psi}_2\right] \\
&-\frac{1}{2}\mathrm{tr}\left[\boldsymbol{K}^{-1}(\boldsymbol{A} - \boldsymbol{A}_0)\boldsymbol{L}^{-1}(\boldsymbol{A} - \boldsymbol{A}_0)^T\right] \\
&-\frac{v_0 + q + 1}{2}\log|\boldsymbol{\Sigma}_\delta| - \frac{1}{2}\mathrm{tr}\left[\boldsymbol{\Psi}_\delta\boldsymbol{\Sigma}_\delta^{-1}\right]
\end{aligned}$$

where $\mathbf{1}_n$ is the n-dimensional vector of 1's.

Since

$$
\begin{aligned}
&\operatorname{tr}\left[\left(\boldsymbol{\eta}-\mathbf{1}_n\boldsymbol{\mu}_{\boldsymbol{\eta}}^T-\mathbb{X}_{\boldsymbol{\mu}_{\mathbf{X}}}\boldsymbol{\Phi}(\boldsymbol{A})\boldsymbol{c}\right)\boldsymbol{\Sigma}_\delta^{-1}\left(\boldsymbol{\eta}-\mathbf{1}_n\boldsymbol{\mu}_{\boldsymbol{\eta}}^T-\mathbb{X}_{\boldsymbol{\mu}_{\mathbf{X}}}\boldsymbol{\Phi}(\boldsymbol{A})\boldsymbol{c}\right)^T\right]\\
=&\operatorname{tr}\left[n\boldsymbol{\Sigma}_\delta^{-1}\left[\bar{\boldsymbol{\eta}}-\boldsymbol{\mu}_{\boldsymbol{\eta}}-\boldsymbol{c}^T\boldsymbol{\Phi}(\boldsymbol{A})^T\left(\bar{\mathbf{X}}-\boldsymbol{\mu}_{\mathbf{X}}\right)\right]\left[\bar{\boldsymbol{\eta}}-\boldsymbol{\mu}_{\boldsymbol{\eta}}-\boldsymbol{c}^T\boldsymbol{\Phi}(\boldsymbol{A})^T\left(\bar{\mathbf{X}}-\boldsymbol{\mu}_{\mathbf{X}}\right)\right]^T\right]\\
&+\operatorname{tr}\left[\boldsymbol{\Sigma}_\delta^{-1}\left(\boldsymbol{\eta}_c-\mathbb{X}_c\boldsymbol{\Phi}(\boldsymbol{A})\boldsymbol{c}\right)^T\left(\boldsymbol{\eta}_c-\mathbb{X}_c\boldsymbol{\Phi}(\boldsymbol{A})\boldsymbol{c}\right)\right]\\
=&n\left[\boldsymbol{\mu}_{\boldsymbol{\eta}}-\boldsymbol{c}^T\boldsymbol{\Phi}(\boldsymbol{A})^T\left(\bar{\mathbf{X}}-\boldsymbol{\mu}_{\mathbf{X}}\right)\right]^T\boldsymbol{\Sigma}_\delta^{-1}\left[\boldsymbol{\mu}_{\boldsymbol{\eta}}-\boldsymbol{c}^T\boldsymbol{\Phi}(\boldsymbol{A})^T\left(\bar{\mathbf{X}}-\boldsymbol{\mu}_{\mathbf{X}}\right)\right]\\
&+\operatorname{tr}\left[\boldsymbol{\Sigma}_\delta^{-1}\left(\boldsymbol{\eta}_c-\mathbb{X}_c\boldsymbol{\Phi}(\boldsymbol{A})\boldsymbol{c}\right)^T\left(\boldsymbol{\eta}_c-\mathbb{X}_c\boldsymbol{\Phi}(\boldsymbol{A})\boldsymbol{c}\right)\right]
\end{aligned}
$$

where $\bar{\boldsymbol{\eta}}=\frac{\mathbf{1}_n^T\boldsymbol{\eta}}{n}$, $\boldsymbol{\eta}_c=\boldsymbol{\eta}-\mathbf{1}_n\bar{\boldsymbol{\eta}}^T$, $\bar{\mathbf{X}}=\frac{\mathbf{1}_n^T\mathbb{X}}{n}$, $\mathbb{X}_c=\mathbb{X}-\mathbf{1}_n\bar{\mathbf{X}}^T$.

Similarly,

$$
\begin{aligned}
&\operatorname{tr}\left[\mathbb{X}_{\boldsymbol{\mu}_{\mathbf{X}}}\left[\boldsymbol{\Phi}(\boldsymbol{A})\boldsymbol{\Delta}\boldsymbol{\Phi}^T(\boldsymbol{A})+\boldsymbol{\Phi}_0(\boldsymbol{A})\boldsymbol{\Delta}_0\boldsymbol{\Phi}_0(\boldsymbol{A})^T\right]^{-1}\mathbb{X}_{\boldsymbol{\mu}_{\mathbf{X}}}^T\right]\\
=&\operatorname{tr}\left[\left[\boldsymbol{\Phi}(\boldsymbol{A})\boldsymbol{\Delta}\boldsymbol{\Phi}(\boldsymbol{A})^T+\boldsymbol{\Phi}_0(\boldsymbol{A})\boldsymbol{\Delta}_0\boldsymbol{\Phi}_0(\boldsymbol{A})^T\right]^{-1}\left[n\left(\bar{\mathbf{X}}-\mathbf{1}_n\boldsymbol{\mu}_{\mathbf{X}}^T\right)\left(\bar{\mathbf{X}}-\mathbf{1}_n\boldsymbol{\mu}_{\mathbf{X}}^T\right)^T+\mathbb{X}_c^T\mathbb{X}_c\right]\right]\\
=&n\left(\boldsymbol{\mu}_{\mathbf{X}}-\bar{\mathbf{X}}\right)^T\left[\boldsymbol{\Phi}(\boldsymbol{A})\boldsymbol{\Delta}\boldsymbol{\Phi}(\boldsymbol{A})^T+\boldsymbol{\Phi}_0(\boldsymbol{A})\boldsymbol{\Delta}_0\boldsymbol{\Phi}_0(\boldsymbol{A})^T\right]^{-1}\left(\boldsymbol{\mu}_{\mathbf{X}}-\bar{\mathbf{X}}\right)\\
&+\operatorname{tr}\left[\boldsymbol{\Delta}^{-1}\boldsymbol{\Phi}(\boldsymbol{A})^T\mathbb{X}_c^T\mathbb{X}_c\boldsymbol{\Phi}(\boldsymbol{A})\right]+\operatorname{tr}\left[\boldsymbol{\Delta}_0^{-1}\boldsymbol{\Phi}_0(\boldsymbol{A})^T\mathbb{X}_c^T\mathbb{X}_c\boldsymbol{\Phi}_0(\boldsymbol{A})\right]
\end{aligned}
$$

Since

$$
\begin{aligned}
&\left[\boldsymbol{\mu}_{\boldsymbol{\eta}}-\boldsymbol{c}^T\boldsymbol{\Phi}(\boldsymbol{A})^T\left(\bar{\mathbf{X}}-\boldsymbol{\mu}_{\mathbf{X}}\right)\right]^T\boldsymbol{\Sigma}_\delta^{-1}\left[\boldsymbol{\mu}_{\boldsymbol{\eta}}-\boldsymbol{c}^T\boldsymbol{\Phi}(\boldsymbol{A})^T\left(\bar{\mathbf{X}}-\boldsymbol{\mu}_{\mathbf{X}}\right)\right]\\
&\left(\boldsymbol{\mu}_{\mathbf{X}}-\bar{\mathbf{X}}\right)^T\left[\boldsymbol{\Phi}(\boldsymbol{A})\boldsymbol{\Delta}\boldsymbol{\Phi}(\boldsymbol{A})^T+\boldsymbol{\Phi}_0(\boldsymbol{A})\boldsymbol{\Delta}_0\boldsymbol{\Phi}_0(\boldsymbol{A})^T\right]^{-1}\left(\boldsymbol{\mu}_{\mathbf{X}}-\bar{\mathbf{X}}\right)\\
=&\left(\boldsymbol{\mu}_{\boldsymbol{D}}-\bar{\boldsymbol{D}}\right)^T\boldsymbol{\Sigma}_{\boldsymbol{D}}^{-1}\left(\boldsymbol{\mu}_{\boldsymbol{D}}-\bar{\boldsymbol{D}}\right)
\end{aligned}
$$

where

$$
\boldsymbol{\mu}_{\boldsymbol{D}}=\begin{bmatrix}\boldsymbol{\mu}_{\mathbf{X}}\\ \boldsymbol{\mu}_\eta\end{bmatrix},\bar{\boldsymbol{D}}=\begin{bmatrix}\bar{\mathbf{X}}\\ \bar{\boldsymbol{\eta}}\end{bmatrix},\boldsymbol{\Sigma}_{\boldsymbol{D}}=\begin{bmatrix}\boldsymbol{\Phi}(\boldsymbol{A})\boldsymbol{\Delta}\boldsymbol{\Phi}(\boldsymbol{A})^T+\boldsymbol{\Phi}_0(\boldsymbol{A})\boldsymbol{\Delta}_0\boldsymbol{\Phi}_0(\boldsymbol{A})^T & \boldsymbol{\Phi}(\boldsymbol{A})\boldsymbol{\Delta}\boldsymbol{c}\\ \boldsymbol{c}^T\boldsymbol{\Delta}\boldsymbol{\Phi}^T & \boldsymbol{\Sigma}_\delta+\boldsymbol{c}^T\boldsymbol{\Delta}\boldsymbol{c}\end{bmatrix},
$$

And

$$
\begin{aligned}
&\operatorname{tr}\left[\boldsymbol{\Sigma}_\delta^{-1}\left(\boldsymbol{\eta}_c-\mathbb{X}_c\boldsymbol{\Phi}(\boldsymbol{A})\boldsymbol{c}\right)^T\left(\boldsymbol{\eta}_c-\mathbb{X}_c\boldsymbol{\Phi}(\boldsymbol{A})\boldsymbol{c}\right)\right]\\
&\quad+\operatorname{tr}\left[\boldsymbol{\Sigma}_\delta^{-1}\left(\boldsymbol{c}-\boldsymbol{M}^{-1}\boldsymbol{\Phi}(\boldsymbol{A})^T\boldsymbol{e}\right)^T\boldsymbol{M}\left(\boldsymbol{c}-\boldsymbol{M}^{-1}\boldsymbol{\Phi}(\boldsymbol{A})^T\boldsymbol{e}\right)\right]\\
=&\operatorname{tr}\left[\boldsymbol{\Sigma}_\delta^{-1}\left[\boldsymbol{c}^T\left(\boldsymbol{M}+\boldsymbol{\Phi}(\boldsymbol{A})^T\mathbb{X}_c^T\mathbb{X}\boldsymbol{\Phi}(\boldsymbol{A})\right)\boldsymbol{c}-2\boldsymbol{c}^T\boldsymbol{\Phi}(\boldsymbol{A})^T\left(\mathbb{X}_c^T\boldsymbol{\eta}_c+\boldsymbol{e}\right)\right]\right]\\
&\quad+\operatorname{tr}\left[\boldsymbol{\Sigma}_\delta^{-1}\left(\boldsymbol{\eta}_c^T\boldsymbol{\eta}_c+\boldsymbol{e}^T\boldsymbol{\Phi}(\boldsymbol{A})\boldsymbol{M}^{-1}\boldsymbol{\Phi}(\boldsymbol{A})^T\boldsymbol{e}\right)\right]\\
=&\operatorname{tr}\left[\boldsymbol{\Sigma}_\delta^{-1}\left(\boldsymbol{c}-\tilde{\boldsymbol{M}}^{-1}\boldsymbol{\Phi}(\boldsymbol{A})^T\tilde{\boldsymbol{e}}\right)^T\tilde{\boldsymbol{M}}\left(\boldsymbol{c}-\tilde{\boldsymbol{M}}^{-1}\boldsymbol{\Phi}(\boldsymbol{A})^T\tilde{\boldsymbol{e}}\right)\right]\\
&\quad+\operatorname{tr}\left[\boldsymbol{\Sigma}_\delta^{-1}\left(\boldsymbol{\eta}_c^T\boldsymbol{\eta}_c+\boldsymbol{e}^T\boldsymbol{\Phi}(\boldsymbol{A})\boldsymbol{M}^{-1}\boldsymbol{\Phi}(\boldsymbol{A})^T\boldsymbol{e}-\tilde{\boldsymbol{e}}^T\boldsymbol{\Phi}(\boldsymbol{A})\tilde{\boldsymbol{M}}^{-1}\boldsymbol{\Phi}(\boldsymbol{A})^T\tilde{\boldsymbol{e}}\right)\right]\\
:=&\operatorname{tr}\left[\boldsymbol{\Sigma}_\delta^{-1}\left(\boldsymbol{c}-\tilde{\boldsymbol{M}}^{-1}\boldsymbol{\Phi}(\boldsymbol{A})^T\tilde{\boldsymbol{e}}\right)^T\tilde{\boldsymbol{M}}\left(\boldsymbol{c}-\tilde{\boldsymbol{M}}^{-1}\boldsymbol{\Phi}(\boldsymbol{A})^T\tilde{\boldsymbol{e}}\right)\right]+\operatorname{tr}\left[\boldsymbol{\Sigma}_\delta^{-1}\tilde{\boldsymbol{H}}\right]
\end{aligned}
$$

where

$$
\begin{aligned}
&\tilde{\boldsymbol{M}}=\boldsymbol{M}+\boldsymbol{\Phi}(\boldsymbol{A})^T\mathbb{X}_c^T\mathbb{X}\boldsymbol{\Phi}(\boldsymbol{A}),\qquad \tilde{\boldsymbol{e}}=\mathbb{X}_c^T\boldsymbol{\eta}_c+\boldsymbol{e},\\
&\tilde{\boldsymbol{H}}=\boldsymbol{\eta}_c^T\boldsymbol{\eta}_c+\boldsymbol{e}^T\boldsymbol{\Phi}(\boldsymbol{A})\boldsymbol{M}^{-1}\boldsymbol{\Phi}(\boldsymbol{A})^T\boldsymbol{e}-\tilde{\boldsymbol{e}}^T\boldsymbol{\Phi}(\boldsymbol{A})\tilde{\boldsymbol{M}}^{-1}\boldsymbol{\Phi}(\boldsymbol{A})^T\tilde{\boldsymbol{e}},
\end{aligned}
$$

From the above equations, we have

$$
\begin{aligned}
&\log p(\boldsymbol{\Sigma}_\delta,\boldsymbol{\mu}_\eta,\boldsymbol{\mu}_{\mathbf{X}},\boldsymbol{\Delta},\boldsymbol{\Delta}_0,\boldsymbol{c},\boldsymbol{A},\boldsymbol{\eta}\mid\mathcal{D})\\
=&-\frac{n}{2}\left(\boldsymbol{\mu}_{\boldsymbol{D}}-\bar{\boldsymbol{D}}\right)^T\boldsymbol{\Sigma}_{\boldsymbol{D}}^{-1}\left(\boldsymbol{\mu}_{\boldsymbol{D}}-\bar{\boldsymbol{D}}\right)\\
&-\frac{1}{2}\operatorname{tr}\left[\boldsymbol{\Sigma}_\delta^{-1}\left(\boldsymbol{c}-\tilde{\boldsymbol{M}}^{-1}\boldsymbol{\Phi}(\boldsymbol{A})^T\tilde{\boldsymbol{e}}\right)^T\tilde{\boldsymbol{M}}\left(\boldsymbol{c}-\tilde{\boldsymbol{M}}^{-1}\boldsymbol{\Phi}(\boldsymbol{A})^T\tilde{\boldsymbol{e}}\right)\right]\\
&-\frac{1}{2}\operatorname{tr}\left[\boldsymbol{\Sigma}_\delta^{-1}(\tilde{\boldsymbol{H}}+\boldsymbol{\Psi}_\delta)\right]-\frac{v_0+q+n+m+1}{2}\log|\boldsymbol{\Sigma}_\delta|\\
&-\frac{1}{2}\operatorname{tr}\left[\boldsymbol{\Delta}^{-1}\left(\boldsymbol{\Phi}(\boldsymbol{A})^T\mathbb{X}_c^T\mathbb{X}_c\boldsymbol{\Phi}(\boldsymbol{A})+\boldsymbol{\Psi}_1\right)\right]-\frac{v_1+m+n+1}{2}\log|\boldsymbol{\Delta}|\\
&-\frac{1}{2}\operatorname{tr}\left[\boldsymbol{\Delta}_0^{-1}\left(\boldsymbol{\Phi}_0(\boldsymbol{A})^T\mathbb{X}_c^T\mathbb{X}_c\boldsymbol{\Phi}_0(\boldsymbol{A})+\boldsymbol{\Psi}_2\right)\right]-\frac{v_2+p-m+n+1}{2}\log|\boldsymbol{\Delta}_0|\\
&-\frac{1}{2}\operatorname{tr}\left[\boldsymbol{K}^{-1}(\boldsymbol{A}-\boldsymbol{A}_0)\boldsymbol{L}^{-1}(\boldsymbol{A}-\boldsymbol{A}_0)^T\right]
\end{aligned}
$$

Note that

$$
\int_{\mathbb{R}^{p+q}}\exp\left[-\frac{n}{2}\left(\boldsymbol{\mu}_{\boldsymbol{D}}-\bar{\boldsymbol{D}}\right)^T\boldsymbol{\Sigma}_{\boldsymbol{D}}^{-1}\left(\boldsymbol{\mu}_{\boldsymbol{D}}-\bar{\boldsymbol{D}}\right)\right]d\boldsymbol{\mu}_{\boldsymbol{D}}=(2\pi n)^{\frac{p+q}{2}}\left|\boldsymbol{\Sigma}_\delta\right|^{\frac{1}{2}}\left|\boldsymbol{\Delta}\right|^{\frac{1}{2}}\left|\boldsymbol{\Delta}_0\right|^{\frac{1}{2}},
$$

and

$$
\begin{aligned}
&\int_{\mathbb{S}^{m\times m}} \exp\left\{-\frac{1}{2}\mathrm{tr}\left[\boldsymbol{\Sigma}_\delta^{-1}\left(\boldsymbol{c}-\tilde{\boldsymbol{M}}^{-1}\boldsymbol{\Phi}(\boldsymbol{A})^T\tilde{\boldsymbol{e}}\right)^T\tilde{\boldsymbol{M}}\left(\boldsymbol{c}-\tilde{\boldsymbol{M}}^{-1}\boldsymbol{\Phi}(\boldsymbol{A})^T\tilde{\boldsymbol{e}}\right)\right]\right\}d\boldsymbol{c}\\
=&(2\pi)^{\frac{mq}{2}}\left|\boldsymbol{\Sigma}_\delta\right|^{\frac{m}{2}}\left|\tilde{\boldsymbol{M}}\right|^{-\frac{q}{2}}\\
=&(2\pi)^{\frac{mq}{2}}\left|\boldsymbol{\Sigma}_\delta\right|^{\frac{m}{2}}\left|\boldsymbol{M}+\boldsymbol{\Phi}(\boldsymbol{A})^T\mathbb{X}_c^T\mathbb{X}\boldsymbol{\Phi}(\boldsymbol{A})\right|^{-\frac{q}{2}}\le(2\pi)^{\frac{mq}{2}}\left|\boldsymbol{\Sigma}_\delta\right|^{\frac{m}{2}}\left|\boldsymbol{M}\right|^{-\frac{q}{2}},
\end{aligned}
$$

We have

$$
\begin{aligned}
&\log\int\int\int p(\boldsymbol{\Sigma}_\delta,\boldsymbol{\mu}_\eta,\boldsymbol{\mu}_{\mathbf{X}},\boldsymbol{\Delta},\boldsymbol{\Delta}_0,\boldsymbol{c},\boldsymbol{A},\boldsymbol{\eta}\mid\mathcal{D})d\boldsymbol{\mu}_{\mathbf{X}}\ d\boldsymbol{\mu}_{\boldsymbol{\eta}}\ d\boldsymbol{c}\\
\le&C_1+\frac{m+1}{2}\log|\boldsymbol{\Sigma}_\delta|+\frac{1}{2}\log|\boldsymbol{\Delta}|+\frac{1}{2}\log|\boldsymbol{\Delta}_0|\\
&-\frac{1}{2}\mathrm{tr}\left[\boldsymbol{\Sigma}_\delta^{-1}(\tilde{\boldsymbol{H}}+\boldsymbol{\Psi}_\delta)\right]-\frac{v_0+q+n+m+1}{2}\log|\boldsymbol{\Sigma}_\delta|\\
&-\frac{1}{2}\mathrm{tr}\left[\boldsymbol{\Delta}^{-1}\left(\boldsymbol{\Phi}(\boldsymbol{A})^T\mathbb{X}_c^T\mathbb{X}_c\boldsymbol{\Phi}(\boldsymbol{A})+\boldsymbol{\Psi}_1\right)\right]-\frac{v_1+m+n+1}{2}\log|\boldsymbol{\Delta}|\\
&-\frac{1}{2}\mathrm{tr}\left[\boldsymbol{\Delta}_0^{-1}\left(\boldsymbol{\Phi}_0(\boldsymbol{A})^T\mathbb{X}_c^T\mathbb{X}_c\boldsymbol{\Phi}_0(\boldsymbol{A})+\boldsymbol{\Psi}_2\right)\right]-\frac{v_2+p-m+n+1}{2}\log|\boldsymbol{\Delta}_0|\\
&-\frac{1}{2}\mathrm{tr}\left[\boldsymbol{K}^{-1}(\boldsymbol{A}-\boldsymbol{A}_0)\boldsymbol{L}^{-1}(\boldsymbol{A}-\boldsymbol{A}_0)^T\right]\\
=&C_1-\frac{1}{2}\mathrm{tr}\left[\boldsymbol{\Sigma}_\delta^{-1}(\tilde{\boldsymbol{H}}+\boldsymbol{\Psi}_\delta)\right]-\frac{v_0+q+n}{2}\log|\boldsymbol{\Sigma}_\delta|\\
&-\frac{1}{2}\mathrm{tr}\left[\boldsymbol{\Delta}^{-1}\left(\boldsymbol{\Phi}(\boldsymbol{A})^T\mathbb{X}_c^T\mathbb{X}_c\boldsymbol{\Phi}(\boldsymbol{A})+\boldsymbol{\Psi}_1\right)\right]-\frac{v_1+m+n}{2}\log|\boldsymbol{\Delta}|\\
&-\frac{1}{2}\mathrm{tr}\left[\boldsymbol{\Delta}_0^{-1}\left(\boldsymbol{\Phi}_0(\boldsymbol{A})^T\mathbb{X}_c^T\mathbb{X}_c\boldsymbol{\Phi}_0(\boldsymbol{A})+\boldsymbol{\Psi}_2\right)\right]-\frac{v_2+p-m+n}{2}\log|\boldsymbol{\Delta}_0|\\
&-\frac{1}{2}\mathrm{tr}\left[\boldsymbol{K}^{-1}(\boldsymbol{A}-\boldsymbol{A}_0)\boldsymbol{L}^{-1}(\boldsymbol{A}-\boldsymbol{A}_0)^T\right]
\end{aligned}
$$

with $\tilde{\boldsymbol{H}}$ is positive semi-definite (1), we get

$$
\begin{aligned}
&\log \int\int\int\int\int\int p(\boldsymbol{\Sigma}_\delta, \boldsymbol{\mu}_\eta, \boldsymbol{\mu}_{\mathbf{X}}, \boldsymbol{\Delta}, \boldsymbol{\Delta}_0, \boldsymbol{c}, \boldsymbol{A}, \boldsymbol{\eta} \mid \mathcal{D}) d\boldsymbol{\mu}_{\mathbf{X}}\ d\boldsymbol{\mu}_{\boldsymbol{\eta}}\ d\boldsymbol{c}\ d\boldsymbol{\Sigma}_\delta\ d\boldsymbol{\Delta}\ d\boldsymbol{\Delta}_0 \\
\leq& C_1 + \frac{(v_0+n-1)q}{2}\log 2 + \log\Gamma_q\left(\frac{v_0+n-1}{2}\right) - \frac{v_0+n-1}{2}\log\left|\tilde{\boldsymbol{H}} + \boldsymbol{\Psi}_\delta\right| \\
&+ \frac{(v_1+n-1)m}{2}\log 2 + \log\Gamma_m\left(\frac{v_1+n-1}{2}\right) - \frac{v_1+n-1}{2}\log\left|\boldsymbol{\Phi}(\boldsymbol{A})^T\mathbb{X}_c^T\mathbb{X}_c\boldsymbol{\Phi}(\boldsymbol{A}) + \boldsymbol{\Psi}_1\right| \\
&+ \frac{(v_2+n-1)(p-m)}{2}\log 2 + \log\Gamma_{p-m}\left(\frac{v_2+n-1}{2}\right) \\
&- \frac{v_2+n-1}{2}\log\left|\boldsymbol{\Phi}_0(\boldsymbol{A})^T\mathbb{X}_c^T\mathbb{X}_c\boldsymbol{\Phi}_0(\boldsymbol{A}) + \boldsymbol{\Psi}_2\right| - \frac{1}{2}\mathrm{tr}\left[\boldsymbol{K}^{-1}(\boldsymbol{A}-\boldsymbol{A}_0)\boldsymbol{L}^{-1}(\boldsymbol{A}-\boldsymbol{A}_0)^T\right] \\
=& C_2 - \frac{v_0+n-1}{2}\log\left|\tilde{\boldsymbol{H}} + \boldsymbol{\Psi}_\delta\right| - \frac{v_1+n-1}{2}\log\left|\boldsymbol{\Phi}(\boldsymbol{A})^T\mathbb{X}_c^T\mathbb{X}_c\boldsymbol{\Phi}(\boldsymbol{A}) + \boldsymbol{\Psi}_1\right| \\
&- \frac{v_2+n-1}{2}\log\left|\boldsymbol{\Phi}_0(\boldsymbol{A})^T\mathbb{X}_c^T\mathbb{X}_c\boldsymbol{\Phi}_0(\boldsymbol{A}) + \boldsymbol{\Psi}_2\right| - \frac{1}{2}\mathrm{tr}\left[\boldsymbol{K}^{-1}(\boldsymbol{A}-\boldsymbol{A}_0)\boldsymbol{L}^{-1}(\boldsymbol{A}-\boldsymbol{A}_0)^T\right] \\
\leq& C_2 - \frac{v_0+n-1}{2}\log|\boldsymbol{\Psi}_\delta| - \frac{v_1+n-1}{2}\log|\boldsymbol{\Psi}_1| - \frac{v_2+n-1}{2}\log|\boldsymbol{\Psi}_2| \\
&- \frac{1}{2}\mathrm{tr}\left[\boldsymbol{K}^{-1}(\boldsymbol{A}-\boldsymbol{A}_0)\boldsymbol{L}^{-1}(\boldsymbol{A}-\boldsymbol{A}_0)^T\right] \\
=& C_3 - \frac{1}{2}\mathrm{tr}\left[\boldsymbol{K}^{-1}(\boldsymbol{A}-\boldsymbol{A}_0)\boldsymbol{L}^{-1}(\boldsymbol{A}-\boldsymbol{A}_0)^T\right],
\end{aligned}
$$

where

$$
\begin{aligned}
C_2 =& C_1 + \frac{(v_0+n-1)q}{2}\log 2 + \log\Gamma_q\left(\frac{v_0+n-1}{2}\right) + \frac{(v_1+n-1)m}{2}\log 2 + \log\Gamma_m\left(\frac{v_1+n-1}{2}\right) \\
&+ \frac{(v_2+n-1)(p-m)}{2}\log 2 + \log\Gamma_{p-m}\left(\frac{v_2+n-1}{2}\right)
\end{aligned}
$$

and

$$
C_3 = C_2 - \frac{v_0+n-1}{2}\log|\boldsymbol{\Psi}_\delta| - \frac{v_1+n-1}{2}\log|\boldsymbol{\Psi}_1| - \frac{v_2+n-1}{2}\log|\boldsymbol{\Psi}_2|
$$

Finally, we have

$$
\begin{aligned}
&\log \int\int\int\int\int\int\int\int p(\boldsymbol{\Sigma}_\delta, \boldsymbol{\mu}_\eta, \boldsymbol{\mu}_{\mathbf{X}}, \boldsymbol{\Delta}, \boldsymbol{\Delta}_0, \boldsymbol{c}, \boldsymbol{A}, \boldsymbol{\eta} \mid \mathcal{D}) d\boldsymbol{\mu}_{\mathbf{X}}\ d\boldsymbol{\mu}_{\boldsymbol{\eta}}\ d\boldsymbol{c}\ d\boldsymbol{\Sigma}_\delta\ d\boldsymbol{\Delta}\ d\boldsymbol{\Delta}_0\ d\boldsymbol{\eta} d\boldsymbol{A} \\
\leq& C_3 + \frac{(p-m)m}{2}\log(2\pi)\frac{m}{2}\log|\boldsymbol{K}| + \frac{p-m}{2}\log|\boldsymbol{L}| \leq \infty.
\end{aligned}
$$

The proof is completed.

Proposition 1 $\tilde{\boldsymbol{H}} = \boldsymbol{\eta}_c^T\boldsymbol{\eta}_c + \boldsymbol{e}^T\boldsymbol{\Phi}(\boldsymbol{A})\boldsymbol{M}^{-1}\boldsymbol{\Phi}(\boldsymbol{A})^T\boldsymbol{e} - \tilde{\boldsymbol{e}}^T\boldsymbol{\Phi}(\boldsymbol{A})\tilde{\boldsymbol{M}}^{-1}\boldsymbol{\Phi}(\boldsymbol{A})^T\tilde{\boldsymbol{e}}$ *is positive semi-definite.*

Proof. Let

$$\boldsymbol{H}_1 = \begin{bmatrix} \boldsymbol{\eta}_c^T\boldsymbol{\eta}_c & \boldsymbol{\eta}_c^T\boldsymbol{\eta}_c\boldsymbol{\Phi}(\boldsymbol{A}) \\ \boldsymbol{\Phi}(\boldsymbol{A})^T\boldsymbol{\eta}_c^T\boldsymbol{\eta}_c & \boldsymbol{\Phi}(\boldsymbol{A})^T\mathbb{X}_c\mathbb{X}_c^T\boldsymbol{\Phi}(\boldsymbol{A}) \end{bmatrix} = \begin{bmatrix} \boldsymbol{\eta}_c^T \\ \boldsymbol{\Phi}(\boldsymbol{A})^T\mathbb{X}_c \end{bmatrix} \begin{bmatrix} \boldsymbol{\eta}_c^T \\ \boldsymbol{\Phi}(\boldsymbol{A})^T\mathbb{X}_c \end{bmatrix}^T,$$

$$\boldsymbol{H}_2 = \begin{bmatrix} \boldsymbol{e}^T\boldsymbol{\Phi}(\boldsymbol{A})\boldsymbol{M}^{-1}\boldsymbol{\Phi}(\boldsymbol{A})^T\boldsymbol{e} & \boldsymbol{e}^T\boldsymbol{\Phi}(\boldsymbol{A}) \\ \boldsymbol{\Phi}(\boldsymbol{A})^T\boldsymbol{e} & \boldsymbol{M} \end{bmatrix} = \begin{bmatrix} \boldsymbol{e}^T\boldsymbol{\Phi}(\boldsymbol{A})\boldsymbol{M}^{-\frac{1}{2}} \\ \boldsymbol{M}^{-\frac{1}{2}} \end{bmatrix} \begin{bmatrix} \boldsymbol{e}^T\boldsymbol{\Phi}(\boldsymbol{A})\boldsymbol{M}^{-\frac{1}{2}} \\ \boldsymbol{M}^{-\frac{1}{2}} \end{bmatrix}^T,$$

therefore, $\boldsymbol{H}_1$ and $\boldsymbol{H}_2$ are both positive semi-definite. Then

$$\begin{aligned} \boldsymbol{H}_1 + \boldsymbol{H}_2 &= \begin{bmatrix} \boldsymbol{\eta}_c^T\boldsymbol{\eta}_c + \boldsymbol{e}^T\boldsymbol{\Phi}(\boldsymbol{A})\boldsymbol{M}^{-1}\boldsymbol{\Phi}(\boldsymbol{A})^T\boldsymbol{e} & \boldsymbol{\eta}_c^T\boldsymbol{\eta}_c\boldsymbol{\Phi}(\boldsymbol{A}) + \boldsymbol{e}^T\boldsymbol{\Phi}(\boldsymbol{A}) \\ \boldsymbol{\Phi}(\boldsymbol{A})^T\boldsymbol{\eta}_c^T\boldsymbol{\eta}_c + \boldsymbol{\Phi}(\boldsymbol{A})^T\boldsymbol{e} & \boldsymbol{M} + \boldsymbol{\Phi}(\boldsymbol{A})^T\mathbb{X}_c^T\mathbb{X}\boldsymbol{\Phi}(\boldsymbol{A}) \end{bmatrix} \\ &= \begin{bmatrix} \boldsymbol{\eta}_c^T\boldsymbol{\eta}_c + \boldsymbol{e}^T\boldsymbol{\Phi}(\boldsymbol{A})\boldsymbol{M}^{-1}\boldsymbol{\Phi}(\boldsymbol{A})^T\boldsymbol{e} & \boldsymbol{\eta}_c^T\boldsymbol{\eta}_c\boldsymbol{\Phi}(\boldsymbol{A}) + \boldsymbol{e}^T\boldsymbol{\Phi}(\boldsymbol{A}) \\ \boldsymbol{\Phi}(\boldsymbol{A})^T\boldsymbol{\eta}_c^T\boldsymbol{\eta}_c + \boldsymbol{\Phi}(\boldsymbol{A})^T\boldsymbol{e} & \tilde{\boldsymbol{M}} \end{bmatrix} \end{aligned}$$

is also positive definite. Since $\tilde{\boldsymbol{M}} = \boldsymbol{M} + \boldsymbol{\Phi}(\boldsymbol{A})^T\mathbb{X}_c^T\mathbb{X}\boldsymbol{\Phi}(\boldsymbol{A})$ is positive definite, the Schur complement of $\tilde{\boldsymbol{M}}$ of $\boldsymbol{H}_1 + \boldsymbol{H}_2$, which is $\tilde{\boldsymbol{H}}$, is positive definite.

S.2.2 Proof of Theorem 2

Proof. (a) ϕ-irreducibility: To establish the ϕ-irreducibility of the proposed Markov Chain, we just need to verify that the proposal densities and acceptance probabilities for updating all parameters are positive. In Algorithm 1, the Metropolis steps always have a positive acceptance probability because both the proposal density function (which is a normal distribution) and the target function are positive. Therefore, the acceptance rate for these steps is always 1. The Gibbs steps can be viewed as Metropolis steps with identical proposal distributions and target distributions, therefore, the acceptance probabilities for the Gibbs steps are always 1 and also positive. The proposal distributions and target distributions in the Gibbs steps include the matrix normal distribution and the Inverse-Wishart distribution, which have positive densities.

(b) Aperiodicity: It can be directly derived from the previous statement and each measurable set on the parameter space with a positive Lebesgue measure can be accessed in a single step from any point.

S.3 Additional Simulation Results

S.3.1 Simulation with non-existent lower dimensional envelope subspace

The proposed BESEM integrates envelope methods to SEM for predictor dimension reduction. Nonetheless, it is important to highlight that in cases where the lower dimensional envelope subspace is non-existent, i.e., when $m = p$, indicating that all covariates are relevant to $\mathbf{Y}$, the BESEM seamlessly simplifies to the standard SEM outlined in Equation (5) without impeding parameter estimation. To demonstrate this specific case, we conduct an additional simulation study with $m = p = 20$, which is exactly the standard SEM setting, and apply the proposed envelope method for estimation and envelope dimension selection. With the candidate envelope dimension of $m = 20$, the BESEM model switches to standard SEM. Figure S6 shows that all three information criterion (IC) values decrease as the candidate envelope dimension m increases, suggesting that BESEM consistently identifies p as the optimal envelope dimension, and it can at least achieve estimation efficiency comparable to the standard SEM approach, regardless of the presence of the envelope subspace.

S.3.2 Sensitivity analyses

Sensitivity to violation of normality assumption. We investigate the robustness of the proposed method to the violation of the normality assumption in the predictor distribution. Specifically, we generate non-normally distributed $\mathbf{X}$ using the "mnor" package in R, with multivariate skewness of 0, 10 and multivariate kurtosis of 415.48 and 1650 for the cases $p = 20, 40$, respectively. Consistent with our previous findings in the normal setups (see, Table 1 and Figure 1), the proposed BESEM method performs stably and demonstrats

improved estimation accuracy in terms of RMSE compared to the standard SEM. Table S4 and Figure S7 present the estimation results for factor loadings and regression coefficients $\boldsymbol{\beta}$, respectively, under two sample sizes, $n = 50$ or 300. Similar patterns are observed for other simulation setups and not explicitly reported here for brevity.

Sensitivity to prior choice. We also explore the robustness of the proposed BESEM to different hyperparameter choices. For this analysis, we employ the configuration with $m = 2$ and $p = 40$ to present the results, as it most closely resembles the real data application to the ADNI dataset. Three different sets of prior choices are considered: (I) $\mathbf{H}_{0,k} = 100\mathbf{I}_{q+1}$, the others remain the same; (II) $\boldsymbol{\Psi}_1, \boldsymbol{\Psi}_2, \boldsymbol{\Psi}_\delta$ are set to 10^{-3} times the identity matrix, the prior covariance matrices for $\boldsymbol{c}$ and $\boldsymbol{A}$ are set to 100 times the identity matrix, and the others stay the same; (III) $\mathbf{H}_{0,k} = 100\mathbf{I}_{q+1}, \boldsymbol{\Psi}_1, \boldsymbol{\Psi}_2, \boldsymbol{\Psi}_\delta$ are set to 10^{-3} times the identity matrix, the prior covariance matrices for $\boldsymbol{c}$ and $\boldsymbol{A}$ are set to 100 times the identity matrix, and the others stay the same. Table S5 shows the estimation results under each scenario based on 100 replications. The performance of BESEM demonstrated overall stability to commonly used prior choices.

Table S1: The bias of elements in $\boldsymbol{\beta}$ in the Simulation, estimated by the proposed BESEM, the standard SEM, and SEM with the BLasso method. Displayed are several randomly selected elements in $\boldsymbol{\beta}$.

(a) $m_{\text{true}} = 2, p = 20$

Method	Envelope $\hat{m}$				Envelope m_{true}				SEM				SEM with BLasso			
n	50	150	300	600	50	150	300	600	50	150	300	600	50	150	300	600
$\beta_{1,1}$	-0.0038	0.0025	-0.0025	0.0047	-0.0084	-0.0073	-0.0004	-0.0035	-0.0069	0.0887	-0.0571	-0.2859	-0.1777	-0.2401	-0.2450	-0.1116
$\beta_{13,1}$	0.0196	0.0035	-0.0101	-0.0001	-0.0083	0.0095	0.0004	0.0045	-0.1245	0.1159	-0.0516	-0.0472	0.4856	0.1211	0.1134	0.0771
$\beta_{7,2}$	0.0101	0.0034	-0.0037	-0.0096	-0.0015	-0.0114	-0.0063	-0.0028	-0.0753	-0.0930	0.0251	-0.3219	-0.4353	-0.1670	0.0017	-0.0406
$\beta_{15,2}$	-0.0186	-0.0097	0.0030	0.0010	0.0006	0.0033	0.0011	0.0026	0.1709	0.0247	0.0493	0.1240	-0.1995	0.1105	-0.1281	0.0642

(b) $m_{\text{true}} = 2, p = 40$

Method	Envelope $\hat{m}$				Envelope m_{true}				SEM				SEM with BLasso			
n	50	150	300	600	50	150	300	600	50	150	300	600	50	150	300	600
$\beta_{5,1}$	-0.0303	0.0201	-0.0004	0.0001	0.0294	0.0092	0.0033	0.0111	-0.0807	0.1157	0.0380	-0.0399	-0.7034	-0.4784	-0.2908	-0.4977
$\beta_{18,1}$	0.0325	-0.0121	0.0013	0.0121	-0.0181	-0.0070	-0.0096	0.0031	0.5396	0.2090	-0.0989	-0.0599	0.1112	-0.3188	-0.0946	-0.0681
$\beta_{30,1}$	0.0121	0.0005	-0.0058	-0.0062	-0.0355	-0.0070	-0.0030	-0.0104	-0.4623	0.0628	-0.1453	-0.0381	0.9250	0.2973	0.3638	0.3989
$\beta_{1,2}$	-0.0114	0.0103	-0.0073	-0.0009	-0.0105	0.0008	-0.0035	0.0001	0.1144	-0.1341	0.0061	0.0582	-0.1140	-0.1482	0.0095	0.0480
$\beta_{25,2}$	-0.0024	-0.0065	-0.0014	-0.0044	-0.0146	-0.0002	-0.0053	-0.0037	0.0876	-0.3007	-0.0536	0.0771	0.1752	0.0753	0.1740	0.2424
$\beta_{32,2}$	-0.0067	-0.0028	-0.0021	0.0029	-0.0250	0.0054	-0.0007	-0.0025	-0.2587	0.0071	-0.1480	-0.0596	-0.4177	-0.3602	-0.2112	-0.1691

Table S2: The computational time (in seconds) of one replication with 16000 iterations in Simulation.

	$p = 20$				$p = 40$			
[width=4em,height=2em]mn	50	150	300	600	50	150	300	600
2	54.96	78.99	119.87	187.06	205.32	279.09	389.34	606.47
4	72.57	105.57	154.52	126.67	265.61	368.43	519.56	816.53
6	82.59	124.92	190.51	312.68	323.67	453.65	642.34	1023.27

Note. The computational time here are the time required for the envelope model to estimate the parameters when the optimal envelope space dimension is determined.

Table S3: Point estimates (Est), standard error estimates (SE), and 95% credible intervals (95% CI) of the elements in $\boldsymbol{\beta}$ by SEM with BLasso method in the ADNI study.

(a) Left hemisphere

ID	Est	SE	95% CI
AmygVol	-0.052	0.056	(-0.1642, 0.0531)
CerebCtx	-0.014	0.089	(-0.1948, 0.1614)
CerebWM	-0.037	0.094	(-0.2347, 0.1433)
**HippVol**	-0.270	0.072	(-0.4166, -0.1319)
InfLatVent	0.030	0.061	(-0.0809, 0.1608)
LatVent	-0.021	0.064	(-0.1571, 0.1033)
EntCtx	-0.071	0.099	(-0.2976, 0.0743)
**Fusiform**	-5.393	1.113	(-7.2579, -3.9678)
InfParietal	0.009	0.045	(-0.0799, 0.0963)
**InfTemporal**	-7.333	1.534	(-9.8934, -5.3704)
**MidTemporal**	-2.502	0.350	(-2.9467, -1.8327)
Parahipp	-0.022	0.072	(-0.1630, 0.1194)
**PostCing**	-2.895	0.384	(-3.4221, -2.1544)
Postcentral	-0.506	0.909	(-2.6434, 0.1815)
**Precentral**	-2.964	0.391	(-3.4925, -2.2238)
Precuneus	-0.053	0.059	(-0.1770, 0.0573)
SupFrontal	-0.009	0.047	(-0.1047, 0.0827)
SupParietal	0.004	0.058	(-0.1123, 0.1241)
SupTemporal	-0.035	0.096	(-0.2694, 0.1368)
**Supramarg**	-2.767	0.916	(-3.7547, -0.6488)
**TemporalPole**	-2.804	0.911	(-3.7657, -0.6631)
MeanCing	0.031	0.045	(-0.0539, 0.1229)
MeanFront	-0.006	0.043	(-0.0953, 0.0789)
MeanLatTemp	-0.066	0.071	(-0.2052, 0.0733)
MeanMedTemp	-0.011	0.064	(-0.1402, 0.1140)
MeanPar	1.598	2.640	(-0.2731, 8.0021)
**MeanSensMotor**	6.904	2.448	(1.1607, 9.4409)
MeanTemp	-0.113	0.208	(-0.6034, 0.1749)

(b) Right hemisphere

ID	Est	SE	95% CI
AmygVol	-0.039	0.055	(-0.1506, 0.0631)
CerebCtx	0.073	0.090	(-0.0877, 0.2648)
CerebWM	0.139	0.101	(-0.0411, 0.3438)
HippVol	0.009	0.069	(-0.1282, 0.1468)
InfLatVent	0.003	0.054	(-0.1064, 0.1115)
LatVent	0.003	0.062	(-0.1151, 0.1272)
**EntCtx**	-0.157	0.067	(-0.2945, -0.0242)
Fusiform	-0.012	0.113	(-0.2656, 0.1810)
InfParietal	0.065	0.047	(-0.0238, 0.1566)
InfTemporal	-0.106	0.158	(-0.4715, 0.1391)
MidTemporal	-0.130	0.089	(-0.3294, 0.0239)
Parahipp	-0.008	0.069	(-0.1498, 0.1258)
PostCing	-0.012	0.085	(-0.1986, 0.1523)
**Postcentral**	-2.354	0.804	(-3.1963, -0.4750)
Precentral	-0.027	0.078	(-0.2100, 0.1097)
**Precuneus**	0.140	0.064	(0.0128, 0.2681)
**SupFrontal**	-0.224	0.051	(-0.3210, -0.1209)
SupParietal	-0.078	0.062	(-0.2062, 0.0368)
**SupTemporal**	-3.022	0.386	(-3.5215, -2.2686)
Supramarg	-0.658	1.042	(-3.0848, 0.1092)
TemporalPole	-0.701	1.062	(-3.1365, 0.1103)
MeanCing	-0.056	0.114	(-0.3006, 0.1474)
**MeanFront**	10.300	1.360	(7.7960, 11.9901)
MeanLatTemp	0.142	0.225	(-0.1699, 0.8008)
**MeanMedTemp**	12.011	2.482	(8.8363, 16.1689)
MeanPar	0.240	0.241	(-0.0974, 0.7920)
MeanSensMotor	0.432	0.421	(-0.1240, 1.4037)
MeanTemp	0.087	0.208	(-0.3240, 0.5245)

Table S4: The root mean squared error (RMSE) and bias (BIAS) of estimated free elements in $\mathbf{\Lambda}$ and some elements of $\boldsymbol{\beta}$ for two cases in Simulation where $\mathbf{X}$ is non-normal distributed.

(a) $m_{\text{true}} = 2, p = 20$, RMSE

Method	Envelope $\hat{m}$		Envelope m_{true}		SEM	
n	50	300	50	300	50	300
$\beta_{1,1}$	0.1144	0.0324	0.0532	0.0431	2.9037	1.0738
$\beta_{13,1}$	0.2146	0.0561	0.1327	0.1030	1.7668	0.6242
$\beta_{7,2}$	0.1010	0.0273	0.0757	0.0592	1.5987	0.5561
$\beta_{15,2}$	0.1005	0.0384	0.0858	0.0584	1.8701	0.5664
λ_1	0.0012	0.0005	0.0015	0.0006	0.0014	0.0006
λ_2	0.0034	0.0015	0.0037	0.0016	0.0038	0.0015
λ_3	0.0052	0.0018	0.0050	0.0021	0.0047	0.0021
λ_4	0.0041	0.0017	0.0045	0.0019	0.0043	0.0019

(b) $m_{\text{true}} = 2, p = 20$, BIAS

Method	Envelope $\hat{m}$		Envelope m_{true}		SEM	
n	50	300	50	300	50	300
$\beta_{1,1}$	0.0067	-0.0028	-0.0030	-0.0261	-0.1096	0.1214
$\beta_{13,1}$	-0.0049	0.0026	-0.0099	0.0143	0.1028	0.0681
$\beta_{7,2}$	-0.0074	-0.0058	-0.0214	-0.0217	-0.2163	0.0248
$\beta_{15,2}$	-0.0246	-0.0015	0.0019	0.0088	0.1652	0.0089
λ_1	-0.0001	0.0001	0.0000	-0.0000	0.0001	0.0000
λ_2	-0.0004	-0.0001	0.0004	0.0003	0.0002	0.0001
λ_3	-0.0004	0.0002	-0.0004	0.0005	0.0000	-0.0001
λ_4	0.0001	-0.0002	0.0001	-0.0003	-0.0003	0.0001

(c) $m_{\text{true}} = 2, p = 40$, RMSE

Method	Envelope $\hat{m}$		Envelope m_{true}		SEM	
n	50	300	50	300	50	300
$\beta_{5,1}$	0.2156	0.0643	0.1753	0.0665	3.3084	0.5886
$\beta_{18,1}$	0.2134	0.0440	0.0771	0.0308	6.7249	1.0627
$\beta_{30,1}$	0.1694	0.0538	0.1176	0.0491	4.6963	0.9579
$\beta_{1,2}$	0.1142	0.0388	0.0924	0.0330	3.8726	0.6143
$\beta_{25,2}$	0.0948	0.0289	0.0869	0.0323	4.1478	0.8145
$\beta_{32,2}$	0.1279	0.0356	0.0924	0.0337	3.1681	0.5567
λ_1	0.0023	0.0011	0.0026	0.0011	0.0021	0.0010
λ_2	0.0027	0.0012	0.0029	0.0012	0.0026	0.0012
λ_3	0.0019	0.0009	0.0021	0.0009	0.0020	0.0009
λ_4	0.0046	0.0018	0.0043	0.0017	0.0037	0.0017

(d) $m_{\text{true}} = 2, p = 40$, BIAS

Method	Envelope $\hat{m}$		Envelope m_{true}		SEM	
n	50	300	50	300	50	300
$\beta_{5,1}$	0.0124	-0.0074	-0.0305	0.0044	0.2721	0.0872
$\beta_{18,1}$	-0.0368	0.0029	-0.0212	0.0010	0.3292	-0.0558
$\beta_{30,1}$	0.0284	-0.0030	-0.0148	-0.0090	1.0440	-0.0541
$\beta_{1,2}$	0.0176	0.0016	-0.0286	-0.0028	-0.1998	0.0339
$\beta_{25,2}$	0.0055	-0.0001	-0.0233	-0.0065	0.3149	0.0107
$\beta_{32,2}$	-0.0058	-0.0008	0.0017	-0.0035	-0.6934	0.0364
λ_1	0.0001	-0.0001	0.0002	-0.0000	0.0002	-0.0000
λ_2	0.0001	-0.0001	0.0002	-0.0001	0.0002	-0.0000
λ_3	0.0002	-0.0000	0.0001	0.0000	0.0002	-0.0000
λ_4	-0.0004	-0.0001	-0.0003	-0.0002	0.0002	-0.0002

Table S5: RMSE and BIAS for some elements of $\boldsymbol{\beta}$ and all the free elements of $\boldsymbol{\Lambda}$ under different priors.

(a) RMSE

Prior	I		II		III	
n	50	300	50	300	50	300
$\beta_{5,1}$	0.1899	0.0752	0.1962	0.0782	0.1959	0.0777
$\beta_{18,1}$	0.0792	0.0320	0.0680	0.0307	0.0736	0.0312
$\beta_{30,1}$	0.1239	0.0480	0.1247	0.0538	0.1189	0.0537
$\beta_{1,2}$	0.0958	0.0376	0.1026	0.0379	0.1042	0.0399
$\beta_{25,2}$	0.0953	0.0365	0.0957	0.0429	0.0990	0.0413
$\beta_{32,2}$	0.0790	0.0398	0.0778	0.0378	0.0803	0.0368
λ_1	0.0025	0.0011	0.0024	0.0011	0.0024	0.0011
λ_2	0.0027	0.0012	0.0026	0.0012	0.0026	0.0012
λ_3	0.0021	0.0008	0.0020	0.0008	0.0021	0.0008
λ_4	0.0042	0.0020	0.0042	0.0017	0.0042	0.0017

(b) BIAS

Prior	I		II		III	
n	50	300	50	300	50	300
$\beta_{5,1}$	0.0224	0.0082	0.0242	0.0100	0.0042	0.0079
$\beta_{18,1}$	-0.0094	-0.0022	-0.0060	-0.0055	-0.0052	-0.0080
$\beta_{30,1}$	-0.0238	-0.0026	-0.0255	-0.0082	-0.0206	-0.0057
$\beta_{1,2}$	-0.0138	-0.0009	-0.0127	0.0007	-0.0208	0.0002
$\beta_{25,2}$	-0.0149	-0.0007	-0.0103	-0.0006	-0.0141	0.0013
$\beta_{32,2}$	-0.0051	-0.0019	-0.0048	-0.0004	0.0017	0.0014
λ_1	0.0002	-0.0000	0.0001	0.0001	0.0001	0.0001
λ_2	0.0003	-0.0003	0.0002	0.0002	0.0001	0.0001
λ_3	-0.0000	0.0000	0.0000	0.0001	-0.0000	0.0001
λ_4	-0.0003	0.0001	-0.0001	-0.0001	-0.0002	-0.0001

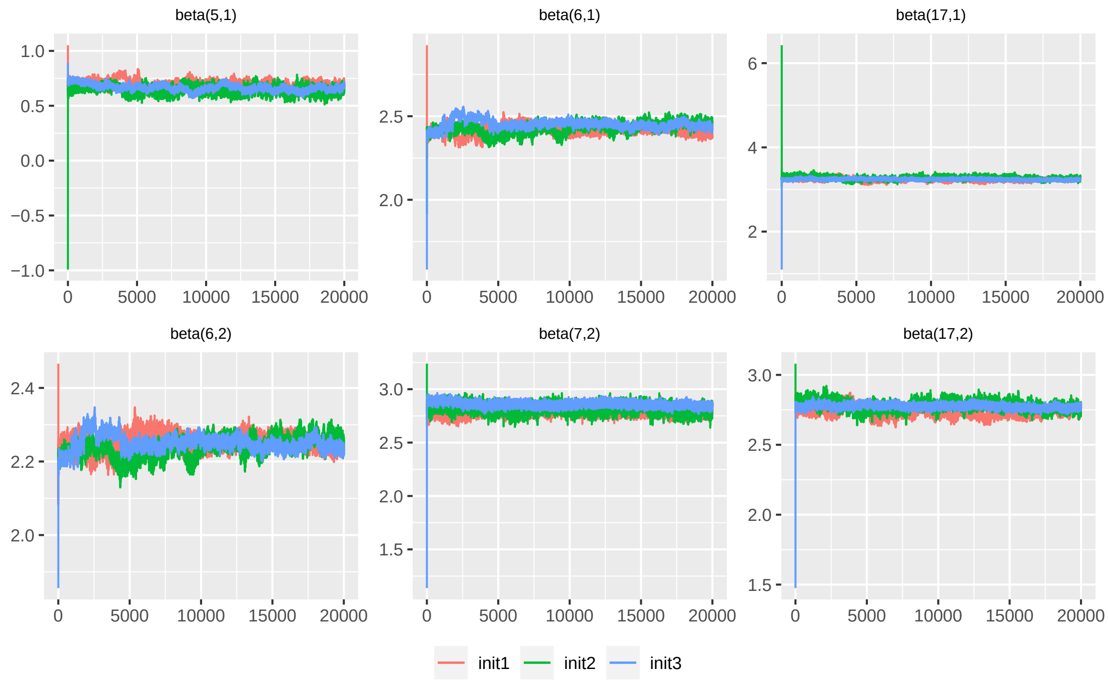

Figure S1: Trace plots of some randomly selected elements of $\boldsymbol{\beta}$ estimated by the proposed BESEM in the simulation study.

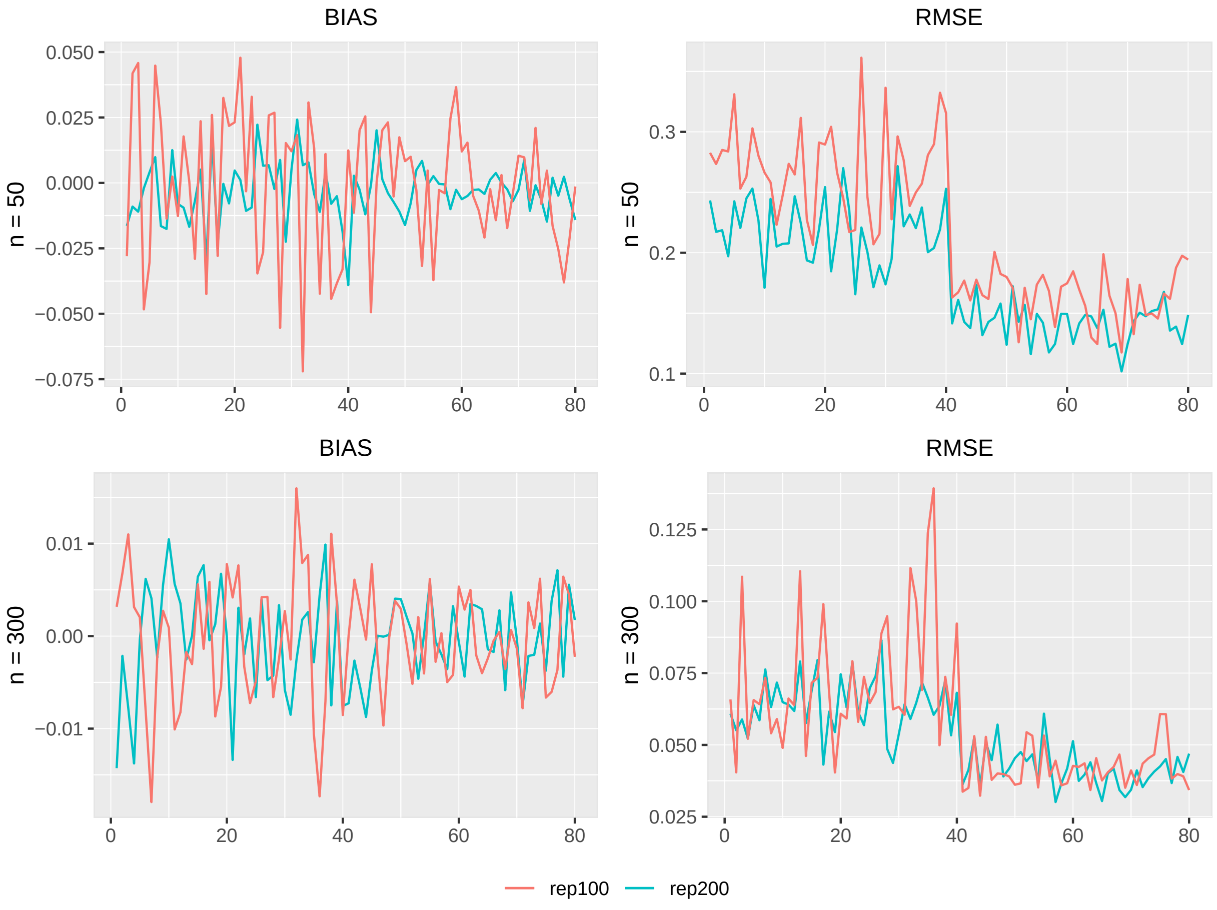

Figure S2: The BIAS and RMSE of the elements in $\boldsymbol{\beta}$ estimated by the proposed BESEM in the simulation scenario with $m = 2$, $p = 40$, and two sample sizes when the analysis is conducted with 100 or 200 replications. x-axis: coordinate of $\boldsymbol{\beta}$. y-axis: value of BIAS or RMSE.

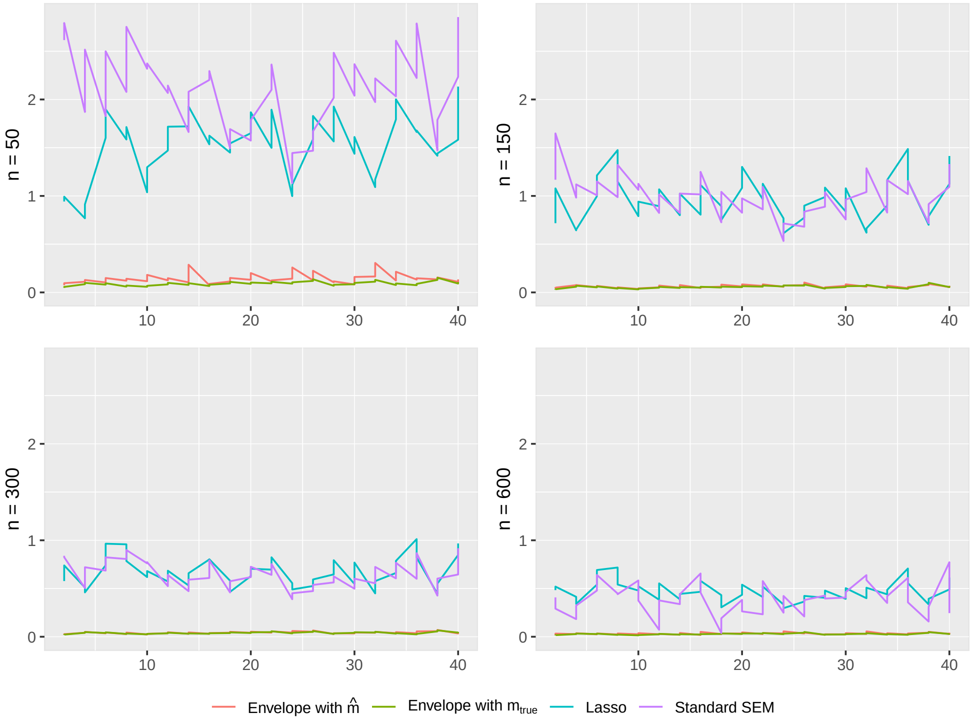

(a) $m_{\text{true}} = 2$ and $p = 20$.

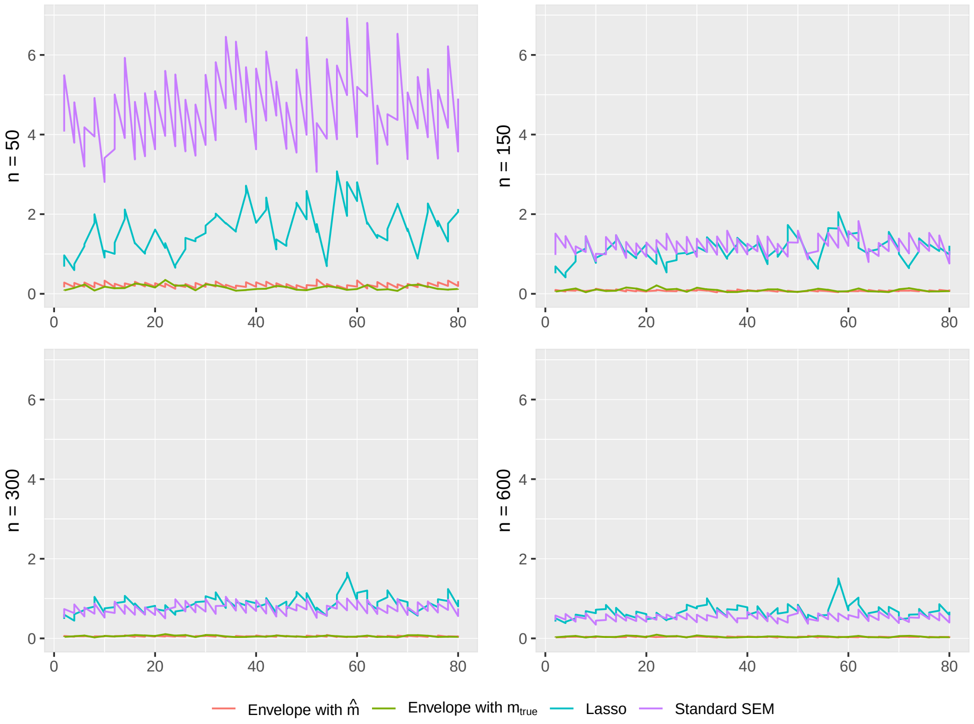

(b) $m_{\text{true}} = 2$ and $p = 40$.

Figure S3: The root mean squared error (RMSE) of the elements of $\boldsymbol{\beta}$ for two cases in Simulation, estimated by the proposed BESEM, the standard SEM, and SEM with the BLasso method. x-axis: coordinate of $\boldsymbol{\beta}$. y-axis: value of RMSE.

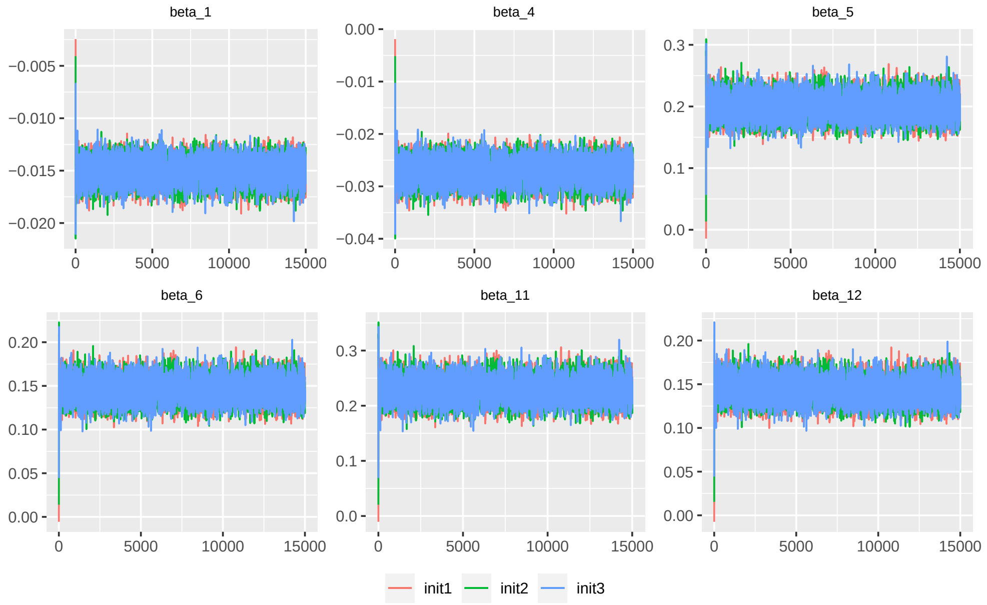

Figure S4: Trace plots of some randomly selected elements of $\boldsymbol{\beta}$ estimated by the proposed BESEM in the analysis of the ADNI dataset.

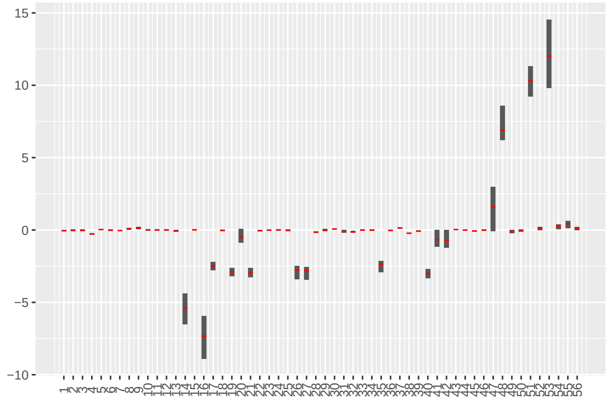

Figure S5: Point and 95% credible interval estimates of each element of $\boldsymbol{\beta}$ for the ADNI study by SEM with BLasso method. x-axis: ID of each ROI, which aligns with the order in Table 4 (adjacent numbers represent the left hemisphere and right hemisphere respectively). y-axis: estimated value. Red short line: the value of the estimated coefficient. Grey rectangle: 95% Credible Interval.

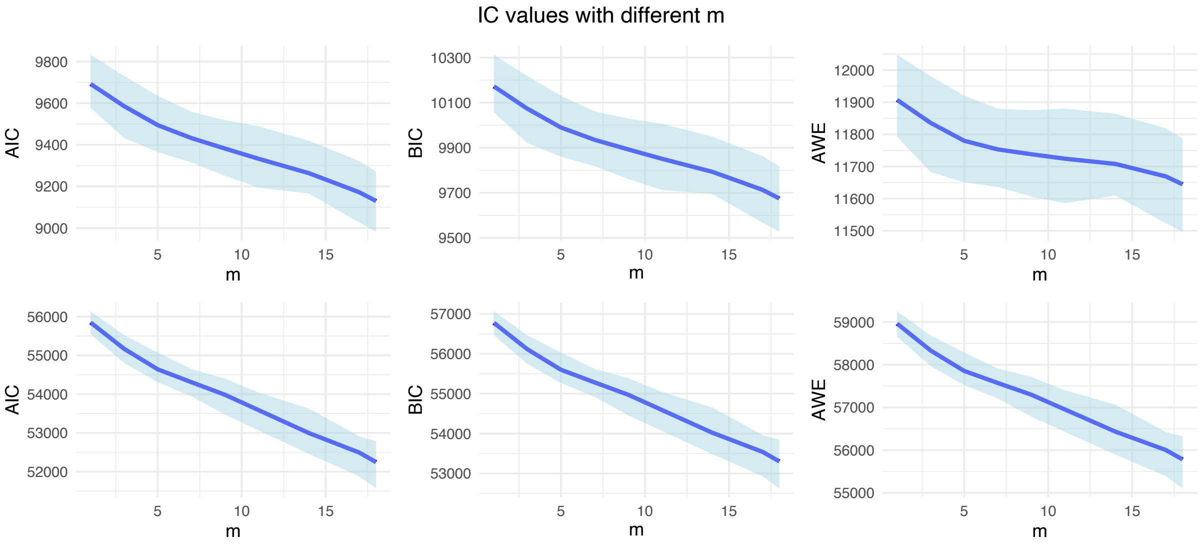

Figure S6: Average IC values and their corresponding 95% quantiles with different m by BESEM method, where $m_{\text{true}} = 20$, $p = 20$. 1st and 2nd rows: $n = 50$ and $n = 300$.

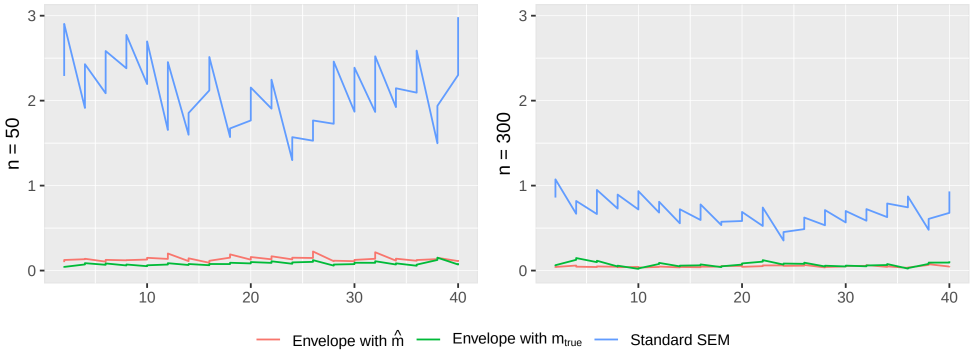

(a) $m_{\text{true}} = 2$ and $p = 20$.

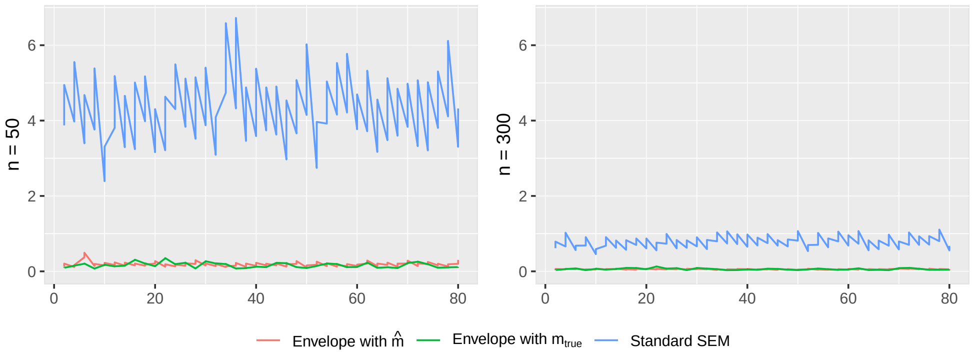

(b) $m_{\text{true}} = 2$ and $p = 40$.

Figure S7: The root mean squared error (RMSE) of the estimated elements of $\boldsymbol{\beta}$ for two cases in Simulation when $\mathbf{X}$ is non-normal. x-axis: coordinate of $\boldsymbol{\beta}$. y-axis: value of RMSE.
